# Revealing patterns of cultural transmission from frequency data: equilibrium and non-equilibrium assumptions

**DOI:** 10.1038/srep39122

**Published:** 2016-12-15

**Authors:** Enrico R. Crema, Anne Kandler, Stephen Shennan

**Affiliations:** 1 University of Cambridge, Department of Archaeology and Anthropology, CB2 3ER, Cambridge,United Kingdom; 2 Max Planck Institute for Evolutionary Anthropology,Department of Human Behavior, Ecology and Culture, 04103,Leipzig,Germany; 3 UCL Institute of Archaeology WC1H 0PY,London, United Kingdom

## Abstract

A long tradition of cultural evolutionary studies has developed a rich repertoire of mathematical models of social learning. Early studies have laid the foundation of more recent endeavours to infer patterns of cultural transmission from observed frequencies of a variety of cultural data, from decorative motifs on potsherds to baby names and musical preferences. While this wide range of applications provides an opportunity for the development of generalisable analytical workflows, archaeological data present new questions and challenges that require further methodological and theoretical discussion. Here we examine the decorative motifs of Neolithic pottery from an archaeological assemblage in Western Germany, and argue that the widely used (and relatively undiscussed) assumption that observed frequencies are the result of a system in equilibrium conditions is unwarranted, and can lead to incorrect conclusions. We analyse our data with a simulation-based inferential framework that can overcome some of the intrinsic limitations in archaeological data, as well as handle both equilibrium conditions and instances where the mode of cultural transmission is time-variant. Results suggest that none of the models examined can produce the observed pattern under equilibrium conditions, and suggest. instead temporal shifts in the patterns of cultural transmission.

Mathematical models of cultural evolution have established a deep understanding of the processes of cultural transmission that are likely to evolve in stable or unstable environments by determining the evolutionary stable strategies of the system under consideration (e.g. in refs 1, 2, 3). Additionally, mathematical modelling has also been instrumental in inferring processes of cultural evolution from empirically observed data. Early works^4^ directly applied principles and methods derived from the neutral theory of molecular evolution, assessing whether observed frequencies of different cultural variants, such as pottery decoration^5^ or baby names^6^, can be distinguished from patterns that might emerge from cultural drift — an equivalent of genetic drift where changes in frequencies are purely the result of sampling error and not the outcome of a selective process. Others (e.g. in ref. 7) have more explicitly explored the balance between selective and stochastic forces. Recent studies^8^,^9^,^10^ have gone beyond this null hypothesis framework, and started to infer which forms of social learning are more likely to have generated observed deviations from this null model, setting as the ultimate goal the discerning of different processes of cultural transmission from empirical frequencies of cultural variants. The complex and iterative nature of the cultural transmission process, however, makes the application of standard statistical inferential frameworks non-trivial, as the likelihood function of most models is analytically intractable. Consequently, selection between competing hypotheses of cultural transmission is often reduced to a comparison of the goodness of fit of specific model predictions to the empirical data (e.g. in refs 9,11, though see^12^ for an exception).

But most of the research mentioned above assumes that the cultural system considered is at equilibrium, expressed by a stationary instantaneous frequency distribution of the variants. The fact that an equilibrium has been reached implies that the parameters affecting the dynamic of the system have been constant for a sufficiently long period of time. However, this might not be a realistic assumption for cultural studies.

Recently, attempts have been made to develop non-equilbrium models of cultural evolution^13^ and to couple those models with Bayesian inference techniques^14^,^15^,^16^. The foundation of this inference framework consists of formulating cultural hypotheses as mathematical models, and deriving those hypotheses that can produce theoretical data similar to observed data using approximate Bayesian computation (ABC; e.g. in ref. 17). The flexibility of this approach is particularly appealing in archaeological research, where smaller sample sizes and reduced chronological resolution due to time-averaging impede the application of most of other existing methods^18^.

The aim of this paper is to examine the changing frequency of pottery motifs from a prehistoric archaeological assemblage under the assumptions that (i) the cultural system producing these frequency was at equilibrium and (ii) certain aspects of the system changed over time. For that we develop an inference framework which is able to 1) integrate a wide range of hypotheses of cultural transmission; 2) tackle the often idiosyncratic and poor chronological resolution of historical data; and 3) accommodate both equilibrium and non-equilibrium assumptions. This set-up allows for an exploration of the effects of equilibrium and non-equilibrium assumptions on the inference of underlying cultural processes.

In more detail, in the equilibrium version of our model we assume that frequencies of different types of pottery vessels are produced by a cultural system characterised by a constant rate of production and transmission mode over a sufficiently long time period, and that their temporal variation between the phases is primarily the result of random drift and archaeological sampling. In the non-equilibrium versions of the model we relax some of these assumptions, either at every point in time or across the different chronological phases identified by the archaeologists. This setting allows us to explore which theoretical framework provides a better correspondence with the observed data, and at the same time identify the most likely hypotheses of social learning between a set of candidates.

## Materials

The data set consists of over 5800 pottery vessels recovered from a small group of settlements of the first farmers in Central Europe in the valley of the Merzbach stream in western Germany. The settlements belong to the so-called *Linearbandkeramik* culture. The number of houses in occupation varied through time, but altogether the settlement and ceramic sequence covers nearly five centuries, from ca. 5300 to 4850 cal B.C. The sites were almost completely excavated prior to their destruction by lignite mining, so the archaeological record of what has survived is largely complete. The ceramic vessels take the form of deep bowls whose decoration is highly distinctive and stylised, comprising a variety of distinct but clearly related band-types that were defined by the original excavation team.

The chronological sequence for the occupation of the Merzbach valley is organised into 15 phases and was created on the basis of a seriation of the pottery assemblages based on motif frequencies^19^ and a detailed stratigraphic and spatial analysis of the sites themselves^20^. These two sequences were correlated with one another (see in ref. 19), a process that involved linking the seriation intervals to the independently defined phases. For the first six phases only two band-types were used with any frequency. All the ceramic assemblages from these phases fall into a single seriation interval because they cannot be distinguished from one another. In the present study these early phases have been excluded (along with phase XV), limiting the scope of the analyses to phases VII to XIV, which covers a temporal interval of c. 180 years^21^. The original catalogue tabulates the data for the Merzbach assemblage in terms of the number of vessels possessing a particular bandtype (one per vessel) by the pit and site in which they were found, and seriation interval in which they occur. For the purposes of analysis the frequencies were first grouped by seriation interval and then the seriation intervals were grouped into the settlement phases or house generations; for each phase the number of houses present was calculated by the excavators^20^. The resulting data analysed here consist of counts of decorative motifs organised into eight phases (VII to XIV) and thirty-six types, with a total 5804 vessels.

The Merzbach dataset has already been been subject of archaeological analyses with the conclusions that the observed changes in frequency are indicative of anti-conformity^22^ and unbiased transmission^23^. Shennan and Wilkinson^22^ compared the observed cultural diversities of each phase to the diversity expected under neutral evolution at equilibrium (see^4^ for details). Neutral evolution assumes that the probability of producing a particular motif in the next time step is proportional to the relative frequency of this motif in the population (i.e. in the assemblage) and that novel motifs are introduced with a constant probability. They used different estimates of effective population size and innovation rate, and showed that in general there was a higher diversity than the model expectation, a result that led them to suggest the presence of an anti-conformist transmission bias — whereby rarer variants have higher chance of being selected compared to the expectations given by the unbiased model. Bentley and Shennan^23^ compared the observed motif frequency distribution with the log-normal distribution expected under a stochastic network growth model of unbiased transmission. The study found a good fit between the data and the hypothesis of unbiased transmission, albeit with the qualification that in the earliest phase there was a higher proportion than predicted of low frequency variants. Both studies use summary statistics to evaluate the correspondence between theory and data and assume that the cultural system producing the different frequency distributions is at equilibrium. Nevertheless they generate different inferences regarding the underlying mechanism of cultural transmission. Additionally, both studies ignore potential effects of time-averaging in archaeological assemblages.

## Methods

### Models of Cultural Change

We describe a general simulation framework aimed at modelling the temporal dynamic of cultural change within a population under different assumptions on population and cultural transmission. Our focus is on historical case studies and therefore we assume the existence of records of changes in the frequencies of cultural variants over time, but note that 1) observed frequencies only describe the composition of a sample and not of the whole population of cultural variants; 2) the frequencies do not represent instantaneous distributions but rather an accumulation (i.e. the time-averaged frequencies) over a certain time period; and 3) that these accumulated frequencies are grouped into a sequence of abutting phases.

More specifically, frequencies of different cultural variants are observed in samples of size *n*_*i*_ at the end of *i *= 1, 2, … consecutive phases of cultural change. Our aim is to produce theoretical samples for all phases conditioned on various assumptions about population and cultural transmission processes that can be compared to the observed samples^15^. For that we assume that the sample size *n*_*i*_ is a fraction 1/*r* of the size of the underlying population *N*_*i*_ of cultural variants. Crucially, the *N*_*i*_ cultural variants at the end of phase *i* are the result of the accumulation of *η*_*i*_ production events during this phase. Each production event represents a combination of cultural transmission and the production of material items that reflect this transmission process, and eventually can become part of the archaeological record. The number of material items being produced during each event is denoted by *v*, so that, with other things being equal, the population (i.e. the assemblage) size at the end of phase *i* is given by N_*i*_ = *η*_*i*_*v*. Estimates of *v* can be archaeologically informed and are influenced by the number of individuals engaged in each production event, i.e. the numbers of social learners in the system. Finally we draw a sample of size *n*_*i*_ randomly from the population of size *N*_*i*_ to obtain a theoretical sample conditioned on a specific hypothesis of cultural change. By simulating each production event, but randomly sampling from a defined sequence of production events we effectively emulate a time-averaging process. Although not implemented here, a more complex sampling process that considers the effect of taphonomic loss can be easily integrated.

**Figure 1:**
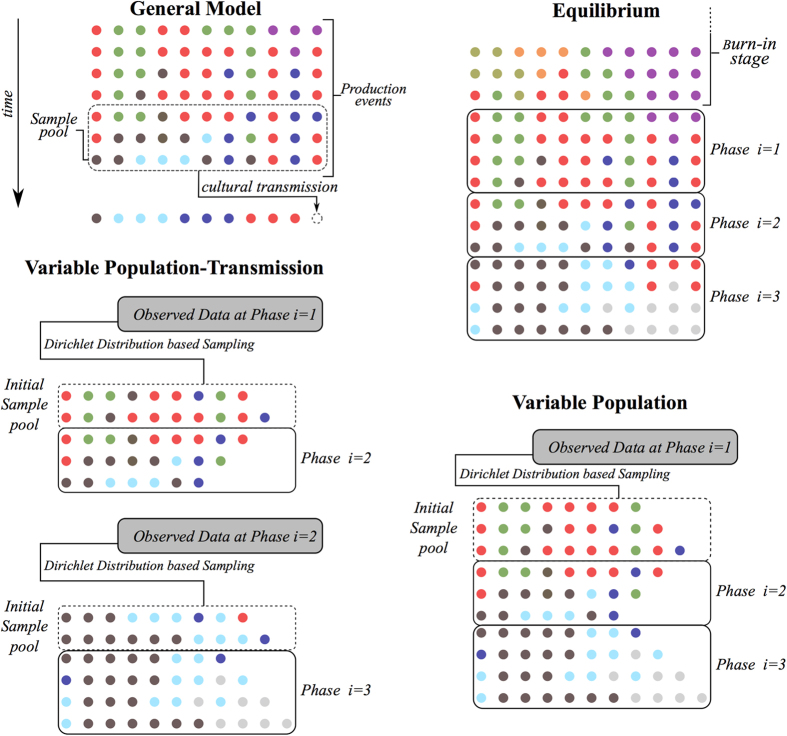
Schematic description of the equilibrium, variable population, and variable population-transmission mode versions of the model. Each coloured dot represent a cultural variant, with each row representing one unit time-step (i.e. production event). The number of dots in each row is defined by the parameter *v*.

The core component of the model is the cultural transmission process specifying which variant types are produced in each production event. More specifically, this process guides an individual’s decision to produce a certain variant type based on the available social information. We assume that social information consists of a sampling pool, composed of the cultural variants of the last *w* production events (see also^24^, and Fig. 1 top-left). In other words, the sampling pool describes the accumulated social information available to the individual and the cultural transmission processes determine the probability with which a specific variant type (in this case a decorative motif) is chosen from this pool. A large number of cultural transmission processes have been identified as acting in human populations (see e.g. in ref. 25); however, in this paper we only focus on unbiased and frequency-dependent transmission processes.

Cultural transmission occurs in an unbiased way if the probability *π*_*j*_ of choosing variant type *j* is proportional to its relative frequency in the sampling pool, denoted by *m*_*j*_ (e.g. in ref. 4). Building on this, frequency-dependent transmission is generally defined by disproportional (compared to unbiased learning) probabilities of copying frequent (conformity) or rare (anti-conformity) variant types^26^. The full spectrum of unbiased, conformist, and anti-conformist transmission can be characterized by the following equation^14^


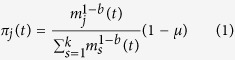


where *k* describes the number of different variant types in the sampling pool, *μ* defines the probability with which a novel variant type is introduced into the system, and *b* describes the strength of frequency-dependent transmission with *b* *<* 0 modelling conformity and *b* *>* 0 anti-conformity. Importantly, for *b* = 0 (1) reduces to unbiased transmission (see SI *Appendix* A and figure S1 for further descriptions of the dynamics of frequency-dependent transmission). Further, (1) implies that with probability 1−*μ* an individual engages in some form of cultural transmission while with probability *μ* it introduces a novel variant into the population through copying error, production error or creative events where novel types are derived from cultural and non-cultural domains outside the system of interest (e.g. introducing a new pottery decoration inspired by the observation of another artefact).

Summarising, during each phase *i* this model set-up produces a an artificial assemblage (i.e. a population) of cultural variants of size *N*_*i*_ conditioned on a specific process of cultural transmission characterised by the model parameters *b*_*i*_, *μ*_*i*_ and *w*_*i*_ (the indices *i* illustrate that these parameters can change between phases). Table 1 lists all variables used in the model, their descriptions and how their values are determined. Based on this set-up we determine in the following the temporal dynamics of the cultural system when assuming that the system is or is not at equilibrium.

### Equilibrium Version

The cultural system is considered to be at equilibrium if the instantaneous frequency distribution, meaning the frequency distribution of cultural variants in each production event, reaches stationarity. To satisfy this condition throughout all phases we assume all model parameters to be constant over time, with the exception of the number of production events associated with each phase (which will be informed by the empirically observed sample size of each phase and reflect inter-phase differences in the productivity of decorated vessels). It follows that the number of cultural variants produced in each production event is constant and there are no changes in the patterns of cultural innovation and learning. Changes in the number of production events effectively capture only the different accumulation of our assemblage data between different phases.

Each simulation is initialised with *v* cultural variants of different types and the values for *μ, b, v, r*, and *w* drawn from prior distributions. Subsequently we perform 5,000 production events. For the first *w* production events the sampling pool naturally has a smaller size but for production events at times larger than *w* only the cultural variants produced in the last *w* events are included. These first 5,000 production events constitute the burn-in phase after which the cultural system reaches stationarity. Following the same procedure, we generate in all consecutive phases assemblages of sizes *N*_*i*_, each resulting from *η*_*i*_ = *N*_*i*_/*v* production events of *v* cultural variants. Lastly, we randomly sample from these assemblages *n*_*i*_ cultural variants (Fig. 1; top-right).

### Variable Population Version

In the next step we relax the assumption that the number of cultural variants generated in each production events is constant over time but still assume that the process of cultural transmission (determined by the parameters *μ, b* and *w*) is the same throughout all phases. Therefore the main differences with the equilibrium version are that 1) *v* is time-dependent, and hence its value can change at each production event; and 2) the model is no longer initialised through a burn-in phase, and instead the initial sampling pool is derived from the observed frequencies. The values *v*(*t*_*i*_ + *τ*), *τ* = 1, …, *η*_*i*_ are inferred from archaeological evidence. under the assumption that the sum of the sequence *v*(*t*_*i*_ + *τ*), *τ* = 1, …, *η*_*i*_ is equal to *N*_*i*_ (see SI *Appendix A* section S1.2 for a detailed derivation). To emulate the initial sample pool we would ideally need information about the distribution of the cultural variants produced by previous production events. This information is, however, not available due to the time-averaged nature of most archaeological records. Following^15^, we use the Dirichlet distribution and generate assemblages of cultural variants from which the observed sample could have been drawn. The initial pool is then generated by randomly sampling *wv*_1_ cultural variants under the constraint that all variant types present in the observed record have to be present. In each subsequent production event *v*_*i*_ cultural variants are produced according to (1) and the sampling pool is updated to include only the latest *w* production events. We note that initially *v*_1_ variants are randomly chosen to be deleted from the sampling pool until each variant in the pool is associated to a chronological marker (so that “older” variants can be identified and removed). This procedure generates variant populations of sizes *N*_*i*_ in each phase *i* and we lastly draw a random sample of size *n*_*i*_ from these assemblages (Fig. 1, bottom-right).

### Variable Population-Transmission Mode Version

Finally we allow both the number of cultural variants produced per production event and the cultural transmission mode, to vary over time. For this we apply the same routine described in the previous section but considering each phase separately (Fig. 1; bottom-left). Thus we generate a sampling pool at the beginning of each phase *i* (following the Dirichlet ansatz, and using the observed frequencies at the end of phase *i*−1,^15^), let *η*_*i*_ production events occur to generate a population of cultural variants at the end of phase *i* conditioned on the cultural transmission process defined by the parameters *b*_*i*_, *μ*_*i*_ and *w*_*i*_ and draw a random sample of size *n*_*i*_. It is worth noting here that we allow *b* to vary between phases but not within each phase. A more complex model, involving a time-series of *b* changing at each production event is theoretically possibly but unfeasible in practice, regardless of the inferential approach being adopted. The only exception would be to use a meta-model defining how *b* can change through time, which would however limit the range of possible sequential values of *b*. Here we chose instead to allow this parameter to vary freely by allowing each phase to be independently assessed.

### Statistical Inference

In the last section we demonstrated how theoretical samples conditioned on specific hypotheses of population and cultural change can be generated. In order to infer which hypotheses are consistent with observed data we apply approximate Bayesian computation (ABC, e.g. in ref. 17). The general idea of ABC is to compute posterior distributions when the likelihood functions of proposed models are not available. In practice this involves a high number of iteration of the following three steps: 1) parameter values are drawn from prior distributions; 2) the error level is measured as the euclidean distance between observed and simulated summary statistics or preferably raw frequency data when it is possible (in the following we base the inference procedure on the absolute frequencies); 3) retain the parameter values of the iterations with the lowest error level. The resulting set of parameters will generate the (joint) posterior distribution which could have produced the observed sample under a given error level (see SI *Appendix B* for more details). The magnitude of the error level provides an indication of how well the model is able to describe the observed data. Additionally, posterior predictive checks (see in ref. 27 for a discussion) provide further insights into the correspondence between theory and data. Posterior predictive checks generate the possible frequency ranges of each individual variant type based on the obtained joint posterior distribution of the model parameters (i.e. through a sufficient number of replications of the simulation framework with model parameters drawn from the joint posterior distribution). The comparison of the observed frequencies with the corresponding prediction intervals allows for conclusions about variant types whose temporal frequency evolution deviates from the expected patterns of the inferred processes of cultural transmission. This knowledge might inform the direction of subsequent analyses (see *Discussion*).

We stress that analyses of sparse data such as those typically found in historical case-studies are likely to reveal the problem of equifinality: a number of underlying cultural processes might be able to produce the same population-level patterns. Broad posterior distributions summarising the parameter values which could produce the observed frequency data up to a small error margin, are indicative of this problem. Nevertheless, even in these situations inference frameworks such as the one described here are instructive as they still narrow down the pool of potential cultural hypotheses.

ABC has been extensively and successfully used in a variety of fields of study (e.g. genetics^28^,^29^,^30^,^31^, epidemiology^32^, biological anthropology^33^, history^34^, and archaeoology^14^,^15^,^16^,^35^) where likelihood functions cannot be determined and proper integration of the uncertainty associated with model assumptions is required. For each version of the model we determine the parameter values which are able to generate variant frequencies similar to the observed ones (i.e. the euclidean distance between theoretical and observed data is smaller than an error level *ε*, see subsection *Calculation of the Error Level ε*) using the rejection algorithm^17^. This algorithm consists of sampling parameter combinations from prior distributions, executing the simulation model, and measuring the euclidean distance *ε* from the observed data (see below). From the pool of *s* iterations of this procedure a proportion α, representing the lowest values of the error level *ε* are retained and the parameter values associated with these are assessed. Here we set *s *= 10^7^ and α = 2 × 10^−5^ for all three versions of the model. Sample codes of the simulation model, the ABC and the dataset can be found on^36^.

### Priors and Constraints

When possible, prior ranges of the model parameters have been informed by ethnographic or archaeological accounts. For example, given that in our model transmission is tightly linked to the production, *v* can be considered both the number of cultural variants produced at each transmission event but also the number of social learners. Hence prior estimates of this parameter have been derived from the observed number of house remains *H*_*i*_ associated to each archaeological phase (see SI *Appendix B* section S2.3 for details) and the parameter *ρ* was introduced as the estimated number of potters per household, with a prior range 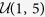. Similarly, the memory parameter *w* was partly informed by ethnographic accounts^37^ on pottery life-span and was drawn from a uniform distribution between 0.2 and 5 years, which was then transformed to number of transmission events assuming a duration of ca 20 years for each archaeological phase (cf.^38^). As no information pertaining to the recovery rate was available we used a fairly wide uninformative range of 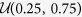. Parameters pertaining to cultural innovation and transmission were also associated with a fairly large prior range (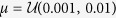 and 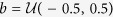 and), albeit the lower threshold of the former was partly informed by the empirical data (so that that there would be a sufficient minimum number of innovation events to generate the observed data), and *b* was limited to a plausible range informed by preliminary test runs of the model.

### Calculation of the Error Level *ε*

The accuracy of the inference results of the ABC methodology depends on the achieved error level *ε* describing the distance between the theoretical and observed samples. Broadly speaking, the posterior distributions obtained describe the parameter ranges which could have produced the observed sample under a given error level *ε* and are only a meaningful approximation of the likelihood function of the generative model if the error level *ε* is sufficiently small^17^. To determine the distance between theoretical and observed samples we use the euclidean distance between corresponding frequency values rather than potentially insufficient summary statistics (as in^22^,^23^). In this way we take full advantage of the observed data.

In the variable population-transmission mode version there is a one-to-one correspondence between the simulated and the empirically observed variants, so the error level *ε* between observed and simulated data can be directly computed as the euclidean distances between matching frequencies. The variable population version also has matching variants, but this applies only for those decorative motifs that exist during the first phase, hence the calculation of *ε* has been limited to these. In the equilibrium version, however, a direct matching is not possible, as the simulation model is initialised without any correspondence to real world data. To overcome this problem and retain, for comparative purposes, the use of raw frequencies for computing *ε* (rather than less informative summary statistics, see SI *Appendix B*) we reordered both the observed and simulated variant types at the beginning of the first phase according to their relative frequency and matched the highest frequency with highest frequency, second-highest with second-highest and so on. We then tracked the frequencies of those variant types present at the beginning of the first phase throughout the remaining phases, and calculated the euclidean distance *ε* between the observed and simulated relative frequencies. In case the number of simulated variants *k*_1_ is higher than the observed 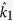 at the first phase, we consider only the 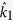 largest frequencies of the simulated data.

In contrast if 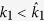 then we assume that the non-modelled 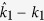 variants have a fixed relative frequency of 0. Focusing on the 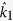 variant types present at the beginning of the first phase allows for a straightforward calculation of the error tolerance and we see this choice justified as in the dataset analysed the sample observed in the last phase is made up to 80% of those 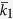 variant types

## Results

The posterior distributions of the inferred parameters obtained from the ABC procedure reveal which parameter values, and therefore which processes (amongst the models considered here), are able to generate theoretical samples similar to the observed samples. From those distributions (see SI *Appendix C* Figs S2–S10) we can conclude that the selection strength *b* is by far the most informative parameter, pointing to a relationship between processes of cultural transmission and observable patterns of cultural change. The posterior distributions of the other model parameters are similar in their shapes and covered in all cases nearly the same range as their respective prior distributions. This indicates how these parameters are less informative, and have smaller influence than *b* in determining the temporal frequencies of cultural variants.

**Figure 2:**
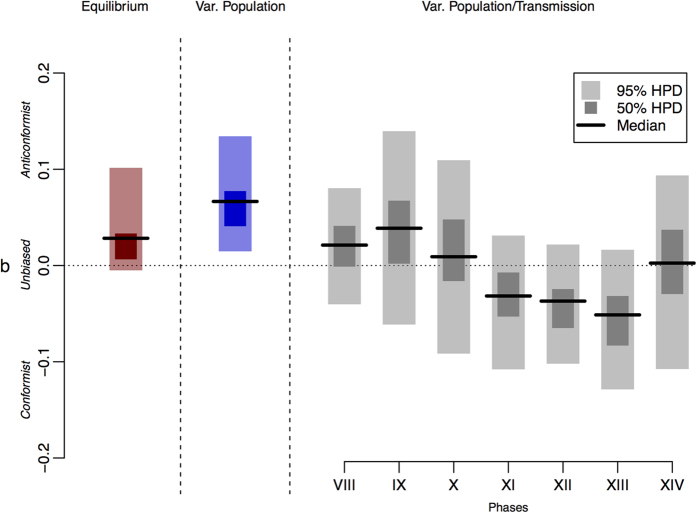
Marginal posterior distributions of *b* for the equilibrium, variable population, and variable population-transmission mode versions.

**Figure 3:**
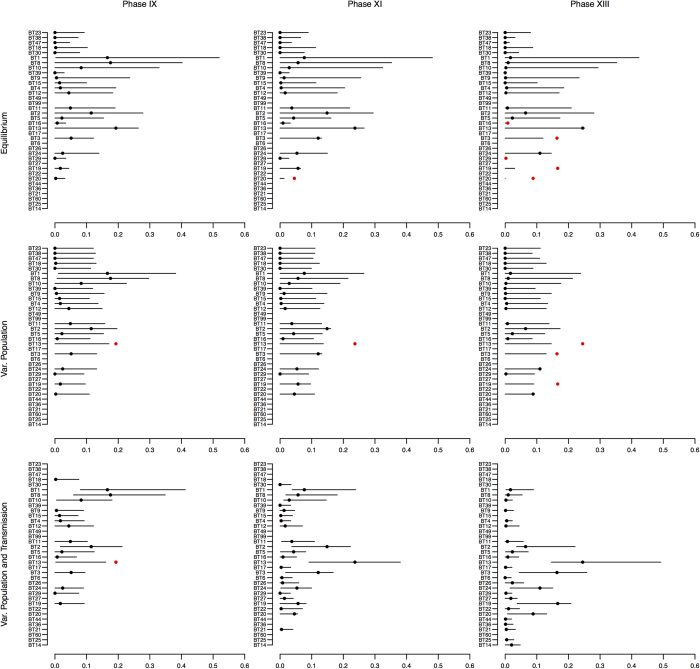
Posterior predictive checks for phase IX, XI and XIII with equilibrium, variable population, and variable population-transmission mode versions of the model. Solid line represent the 95% HPDI predicted by the model while the dots show the observed frequencies (black filled when within the 95% HPDI and red filled when outside this range).

In the equilibrium version of the model, the median posterior density of *b* was 0.028 which would suggest the presence of some degree of weak anti-conformist bias, albeit the 95% highest posterior density interval (HPDI) covers a range between −0.005 and 0.102 (see Fig. 2). The variable population version of the model appears to show a stronger support of an anti-conformist bias, with the entire 95% HPDI (0.015–0.134) larger than zero and a median value of 0.066. The posterior predictive check of both the equilibrium and the variable population versions shows, however, a comparatively poor performance (Fig. 3, see also SI, Figs S11–S13), as well as higher error levels (Fig. S14). This seems to be particularly the case for later phases, where for both models the 95% predicted ranges of the different cultural variants fail to capture the increase in the observed frequencies of several variants (e.g. BT3, BT19, BT13; see SI *Appendix C* Figs S11–S12). This suggests that neither equilibrium conditions, nor a changing number of social learners can explain the observed data, and it hints at the possibility that a change in the process of cultural transmission might have occurred. In other words, the equilibrium version and the variable population version are not able to closely resemble the observed data in all phases, and in particular to capture the increase in the frequencies of several variants starting from around phase X (see SI *Appendix C* Figs S11–S12). Given that the best fitted parameter combinations of the equilibrium and variable population version suggest an anti-conformist bias, the posterior predictive check generally shows a dynamic where rare variants are initially selected and once they become more common their selective advantage diminishes. It follows that the continuous increase in frequency exhibited by variants such as BT19 cannot be achieved by a fixed anti-conformist bias.

This idea is supported by the results of the analysis of the variable population-transmission mode version (see Fig. 2). The posterior distributions of *b* for the individual phases suggest fluctuations (although the 95% HPDI of *b* is not sufficiently narrow to fully dismiss competing models), with earlier stages (phase VIII–X) showing stronger support for an anti-conformist bias and intermediate phases (XI–XIII) for a conformist bias (see SI *Appendix C* section S3.3 for more analysis of the areas of overlap of the posterior distributions). These changes in transmission mode would initially favour rare variants, but even as they become increasingly common their frequency dependent selective advantage will not decline thanks to a shift to a more conformist transmission bias. The posterior predictive check of the variable population-transmission mode version shows indeed a clear improvement (Fig. 3; see also SI *Appendix C* Fig. S13), with the 95% range of the model prediction including all observed frequencies except three variants —BT13 during phase IX, and to a lesser extent BT19 at phase XII and BT 14 at phase XIV–. In all cases, observed frequencies are higher than what is expected by the best fit model.

## Discussion

Archaeological data represent both a challenge and an opportunity for cultural evolutionary studies. Comparatively smaller sample sizes, time-averaging, and other qualitative and quantitative constraints do not ease the direct applications of many of the techniques developed for other contexts. At the same time, the possibility to investigate cultural dynamics at a larger temporal scale broadens the field of study with new perspectives and questions.

In this paper we used a statistical inference framework to overcome problems that are typically encountered in the archaeological record. Issues such as time-averaging, or the integration of independent variables that might influence the change in the temporal frequency of cultural variants have been tackled through the development of a simulation model. The most relevant conclusion showcased by our study is the inability of a fixed mode of cultural transmission to produce the observed changes in the frequency of decorative motifs found in the Merzbach assemblage. To better capture these changes we need to allow for temporal changes in the patterns of cultural transmission over time. These results highlight a potential issue shared by many models of cultural evolution designed to analyse the individual- and/or population-level consequences of cultural transmission. Can we rest on the assumption that the cultural system under consideration is at equilibrium? In situations where sparse frequency data (e.g. as provided by the archaeological record) is the only available empirical evidence how strongly does the equilibrium assumption affect our ability to infer underlying processes of cultural evolution? Our results indicate that equilibrium should be an hypothesis to be tested, rather than an assumption to be hold *a priori*.

The number of studies exploring cultural frequency data is too small — and they are too diverse in their scale and methods — to establish a rule of thumb for determining when equilibrium assumptions can be valid and when not. By evaluating the performance of the best-fit model under equilibrium assumptions, we illustrated a way to determine whether a system in equilibrium, under the specific assumptions of candidate models, is capable of producing the observed frequencies. In the case of the Merzbach assemblage, the most likely transmission mode under equilibrium assumptions — an anti-conformist bias — failed to capture the changing frequencies of several cultural variants. In one sense this is hardly surprising; demographic and cultural processes driving cultural change are unlikely to remain constant over longer temporal spans, making long-term historical data particularly unsuited to equilibrium assumptions. We note, however, that analyses at a short temporal scale are not necessarily immune to the problem, as a sufficient amount of time is required for the cultural system to reach the innovation-drift equilibrium (although this depends on the specific mode of transmission).

Posterior predictive checks generate the possible frequency ranges based on the posterior distribution of the model parameters and provided an additional assessment of the quality of the inference obtained^27^. In our case studies those checks show how temporally constant processes of cultural transmission are unable to produce the steady increase in variant frequencies observed for a number of the variants in the Merzbach assemblage.

Figures S11 and S12 in the SI show that the very slow increase in frequency of certain variant types in the later phases (e.g. BT3, BT13, BT19, BT20) together with the frequency patterns of the remaining variants cannot be replicated by assuming the same transmission process in all phases. This might be indicative of an additional selective advantage of those motifs not captured by the proposed model which is potentially specific to the motif itself, or of changing processes of cultural transmission between phases. We explored the latter hypothesis and concluded that earlier phases possess a tendency towards anti-conformity while later phases possess a tendency towards conformity (see Fig. 2). We note, however, that in phases IX, XII and XIV one of the observed frequencies was outside the predictive intervals, suggesting that a frequency-biased transmission with the range of parameters explored here is not able to produce the observed record for those phases. Predictive checks thus enables the exploration of instances where (meaning in which variant type) deviations between theory and data take place and therefore might inform further, detailed analyses about the origins of these deviations.

The method has, however, also revealed some limitations typically imposed by the nature of most archaeological data. Firstly, in order to perform a non-equilibrium analysis at least two data points have to be available. Additionally, the accuracy of analyses will likely be dependent on the accuracy of the estimate of the temporal distance between the data points^13^. Secondly, while the underlying model of cultural change can mimic the effects of time-averaging for its output, it requires the best estimate of the initial sampling pool of the system under analysis. Using the Dirichlet approach generates populations from which the observed sample could have been drawn with a positive probability. However, as the sample is subject to time-averaging the generated populations will likely overestimate the presence of older variant types which might be already extinct. It is worth noting that for coarse grained chronologies (meaning for relatively long temporal distances between data points) the non-equilibrium inferential framework proposed here might well become increasingly similar to the equilibrium version, as the simulated process would reach equilibrium properties in its output. Lastly, the inference framework presented here includes only transmission processes that can be described by (1), and hence does not consider alternative formalisations of frequency biased transmission (see e.g. in ref. 11) nor other forms of cultural transmission (e.g. in ref. 25). This is certainly not a problem limited to the assessment of the archaeological or historical record, but appropriate mathematical formalisations of alternative processes in the form of copying probabilities will allow for a straightforward generalisation of this framework.

To conclude, our attempt at inferring patterns of cultural transmission in the Merzbach assemblage has revealed a cultural system that is unlikely to be in equilibrium conditions. Instead our approach hints at the possibility of shifts between anti-conformist and conformist modes of social learning. While the inference framework developed in this paper requires further testing with simulated data in controlled conditions, we argue that the challenge posed by archaeological case studies can contribute to methodological and theoretical advances in the field of cultural evolutionary studies.

## Additional Information

**How to cite this article**: Crema, E. R. *et al*. Revealing patterns of cultural transmission from frequency data: equilibrium and non-equilibrium assumptions. *Sci. Rep.* **6**, 39122; doi: 10.1038/srep39122 (2016).

**Publisher's note:** Springer Nature remains neutral with regard to jurisdictional claims in published maps and institutional affiliations.

## Supplementary Material

Supplementary Information

## Figures and Tables

**Table 1:** Model parameters and their description.

Variable	Description
POPULATION PARAMETERS	
*n*_*i*_	Sample size at phase *i* [observed]
*r*_*i*_	Sample recovery rate at phase *i* [inferred]
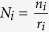	Size of the population of cultural variants in phase *i* [calculated]
*v*(*t*_*i*_)	Number of cultural variants produced at each transmission event *t* within phase *i* [inferred & calculated; see SI]
*η*_*i*_	Number of transmission/production events during phase *i* [calculated]
CULTURAL TRANSMISSION PARAMETERS	
*μ*_*i*_	Innovation rate of phase *i* [inferred]
*b*_*i*_	Strength and sign of frequency-dependent transmission in phase *i* [inferred]
*w*_*i*_	Number of production events that provide social information in phase *i* [inferred]
*π*_*j*_(*t*)	Probability of generating variant type *j* at time *t* [calculated] (see equation 1)
