# Supplementary material for: Revealing patterns of cultural transmission from frequency data: equilibrium and non-equilibrium assumptions
Source: Sci Rep. 2016 Dec 15;6:39122. doi: 10.1038/srep39122 (PMC5156924; doi:10.1038/srep39122)
Supplement: Supplementary Information [file srep39122-s1.pdf]

SUPPLEMENTARY INFORMATION
for
Revealing patterns of cultural transmission from frequency data: equilibrium and non-equilibrium assumptions

Enrico Crema, Anne Kandler, Stephen Shennan

S1 Appendix A - Model of Cultural Change

In this section we provide a detailed description of the simulation framework presented in the main text. We utilise all archaeological information available for the considered episode of cultural change described by the Merzbach assemblage. This assemblage consists of several phases i, $i = 1, \dots, T$ and records the frequencies of different cultural variant types (in the form of decorated pottery motifs) in the archaeological record attributed to these phases. Each phase i lasts from time t_i to time t_{i+1} and is characterized by the sample $\bar{S}(t_{i+1}) = [\bar{s}_1(t_{i+1}), \dots, \bar{s}_{\bar{k}_{i+1}}(t_{i+1})]$ of size $\bar{n}_i$ accumulated throughout the phase. The variables $\bar{s}_j$ describe the absolute frequencies of cultural variant type j in the sample and $\bar{k}_{i+1}$ denotes the number of cultural variants present in the sample at the end of the phase t_{i+1}. Additionally, it is known how many households, denoted by $\bar{H}(t_i)$, contributed to the production of cultural variants at time point t_i.

We assume that each phase i consists of η_i production events. In each production event all present households produce ρ_i cultural variants (ρ_i can be interpreted as the number of potters per household) which results in $\nu_i = \rho_i \bar{H}(t_i)$ cultural variants in total. This means that η_i production events together generate the population of cultural variants at time t_{i+1}, the end of the phase. The size of the population at t_{i+1} is determined by $N_i = \frac{1}{r_i}\bar{n}_i$ where the variable r_i describes the fraction of the population that is recovered with the observed archaeological record of size $\bar{n}_i$. With these assumptions we introduce a "cultural" time scale specifying the number of transmission events, η_i, that are needed to generate a population of size N_i at the end of the phase. Our modelling framework is thus independent of the estimation of the duration of each phase.

To model the process of cultural change we need to determine the variant types that are produced in each of the η time steps (As the same logic applies throughout all phases we omit in the following the index i.). We assume that cultural change is primarily dictated by a combination of cultural transmission processes and innovation. In more detail, cultural transmission processes guide an individual's decision to produce a certain variant type based on the available social information. We assume that an individual can select a variant type from a sampling pool, which is composed of the cultural variants of the w last production events. In other words, the sampling pool describes the accumulated social information available to the individual and the cultural transmission processes determine the probability with which a specific variant type is selected from the sample pool. A large number of cultural transmission processes have been identified to act in human populations (see e.g. [1]) , however, here we only focus on unbiased and frequency-dependent transmission processes.

Cultural transmission occurs in an unbiased way if the probability π_j of selecting variant type j is proportional to its relative frequency in the sample pool, denoted by m_j. It holds

$$\pi_j(t) = \frac{m_j(t)}{\sum\limits_{s=1}^{k} m_s(t)}(1-\mu) \tag{1}$$

where k the number of different variant types in the sampling pool and μ defines the probability with which a novel variant type is introduced into the system (see e.g. [2]).

Frequency-dependent transmission, in form of conformity and anti-conformity, is defined as the disproportionate copying of frequent and rare variant types, respectively [3] and it holds

$$\pi_j(t) = \frac{m_j(t)^{1-b}}{\sum_{s=1}^{k} m_s(t)^{1-b}}(1-\mu) \tag{2}$$

[4]. Here, the parameter b describes the strength of frequency-dependent transmission with $b < 0$ modelling conformity and $b < 0$ anti-conformity. We note that for $b = 0$ Eq. (2) reduces to the copying probability of unbiased transmission (i.e. Eq. (1)). Consequently, we assume that with probability $1 - \mu$ an individual engages in some form cultural transmission while with probability μ it introduces a novel variant into the population through copying error, production error or creative events where novel traits are derived from cultural and non-cultural domains outside the system of interest (e.g. introducing a new pottery decoration inspired from the observation of another artefact).

By means of a toy example we demonstrate the dynamic of frequency-dependent bias. If we assume $k = 5$ cultural variants with the relative frequencies $m_1 = 0.6$, $m_2 = 0.2$, $m_3 = 0.1$, $m_4 = 0.05$ and $m_5 = 0.05$ and no innovation, i.e. $\mu = 0$, π_1 will be equal to m_1 for unbiased learning ($b = 0$), $\pi_1 > m_1$ for conformist bias (e.g. $\pi_1 = 0.637$, for $b = -0.1$) and $\pi_1 < m_1$ for anti-conformist bias (e.g. $\pi_1 = 0.56$, for $b = 0.1$). Larger or smaller values of b will determine stronger conformism or anti-conformism, whilst values close to 0 will produce results similar to unbiased transmission. In this situation unbiased transmission and conformity result in stationary distributions where only one cultural variant is present in the population while anti-conformity produces a stationary distribution where all variants possess the same frequency. The magnitude of b determines how quickly those distributions are reached.

Fig. S1 illustrates this concept. Different values of b showcase different rates by which the system converges to the two stationary distributions; here measured using Pielou's evenness index given by

$$d_{\text{Pielou}} = -\frac{\sum_{s=1}^{k} m_s \ln m_s}{\ln k}$$

which yields 1 when all frequencies are identical and 0 when one variant has a frequency of 1.

Summarizing, cultural change is modeled by performing the following two steps in each of the η_i time steps:

i. Generate $\nu(t_i)$ cultural variants according to equation (2) and update the aggregated population of cultural variants accordingly.

ii. Replace the oldest $\nu(t_i)$ cultural variants in the sampling pool with the ones generated in step i.

Consequently, during each phase i this model set-up produces a population of cultural variants of size N_{i+1} conditioned on a specific process of cultural transmission characterized by the model parameters b_i, μ_i and w_i. To produce a theoretical sample at time point t_{i+1} (i.e. at the end of the phase) we randomly draw $\bar{n}_i$ cultural variants from this population. Tab. S1 lists all variables used in the model, their descriptions and how their values are determined.

In the following we describe how the dynamic of the cultural system can be modelled when assuming that the system is or is not at equilibrium.

S1.1 Equilibrium version

The cultural system is considered to be at equilibrium if the frequency distribution of a population of cultural variants, generated by consecutive production events, reaches stationarity. To satisfy this condition

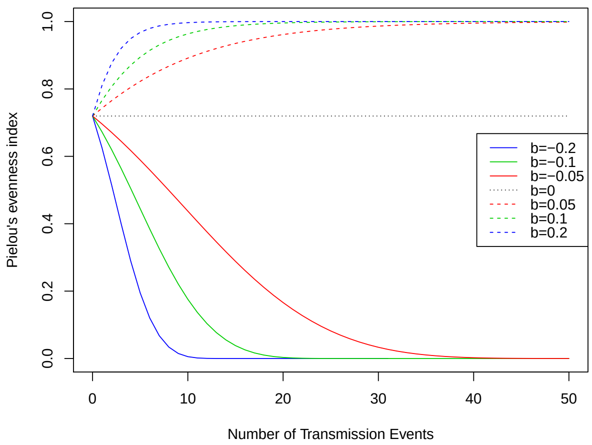

Figure S1: Relationship between the number of transmission events and Pielou's evenness index d_{Pielou} for $b = -0.2, -0.1, -0.05, 00.05, 0.1, 0.2$ and $\mu = 0$. Initial frequencies of the five variant types are $m_1 = 0.6$, $m_2 = 0.2$, $m_3 = 0.1$, $m_4 = 0.05$ and $m_5 = 0.05$.

throughout all phases we assume all model parameters to be constant over time (i.e. $\mu_i = \mu$, $b_i = b$, $\bar{H}(t_i) = \bar{H}$, $\rho_i = \rho$, $r_i = r$, $w_i = w$ for all phases i). This means that there are no population differences between the phases (i.e. the number of cultural variants generated at each production event is constant) and the same process of cultural transmission is acting. We assume the existence of $\bar{H} = \frac{1}{T}\sum_{i=1}^{T} \bar{H}(t_i)$ households in each phase.

Each simulation is initialized with $\nu = \rho\bar{H}$ cultural variants of different types and the values for μ, b, ρ, r, and w are drawn from prior distributions (for details see section S2.3).

Subsequently we perform a 5,000 production events according to steps i. and ii. described above. This constitutes the burn-in phase after which the cultural system is at equilibrium. We note that for the first w production events the sampling pool naturally has a smaller size but for production events at times larger than w only the cultural variants produced in the last w events are contained in the sampling pool. Following the same procedure, we generate $T - 1$ consecutive populations of size N_i (i.e. each population is the aggregation of

$$\eta_i = \frac{N_i}{\rho\bar{H}} \tag{3}$$

production events of ν cultural variants). Lastly we sample $\bar{n}_i$ cultural variants from each population at the end of phase i so that the theoretical samples generated conditioned on the assumed transmission process (characterized by the values of μ, b and w) can be compared to the observed sample.

S1.2 Variable Population Version

In the next step we allow for temporal variations in number of cultural variants produced at each transmission event but still assume that the process of cultural transmission (determined by the parameters μ, b, w) is the same throughout all phases. Additionally, we no longer initialise the model through a burn-in period but use the observed frequencies at the beginning of phase 1 to emulate the initial sample pool. To do so we would ideally need information about the distribution of the cultural variants produced by previous

Variable	Description
POPULATION PARAMETERS	
n_i	Sample size at phase i [observed]
r_i	Sample recovery rate at phase i [inferred]
$N_i = \frac{n_i}{r_i}$	Size of the population of cultural variants in phase i [calculated]
$\bar{H}(t_i)$	Number of households at time t_i [observed]
ρ_i	Number of cultural variants produced per household in each production event of phase i [inferred]
$\nu(t_i+\tau) = \rho_i \bar{H}(t_i+\tau)$	Number of cultural variants produced at each transmission event t within phase i [calculated]
η_i	Number of transmission/production events during phase i (see Eqs. (3) and (5)) [calculated]
CULTURAL TRANSMISSION PARAMETERS	
μ_i	Innovation rate of phase i [inferred]
b_i	Strength and sign of frequency-dependent transmission in phase i [inferred]
w_i	Number of production events that provide social information in phase i [inferred]
$\pi_j(t)$	Probability of generating variant type j at time t (see Eq. (2)) [calculated]

Table S1: Model parameters and their description.

production events. This information is, however, not available. Following [5], we use the Dirichlet distribution ansatz instead and generate populations of cultural variants, $\mathbf{P}(t_1)$, from which the observed sample, $\bar{S}(t_1)$ could have been drawn with a positive probability by sampling from the unnormalized posterior distribution

$$P(\mathbf{P}|\bar{S}) \propto P(\mathbf{P})P(\bar{S}|\mathbf{P}) \propto R_1^{\alpha_1-1+\bar{m}_1} R_2^{\alpha_2-1+\bar{m}_2} \cdot \ldots \cdot R_{\bar{k}_1}^{\alpha_{k_1}-1+m_{k_1}} R_{\bar{k}_1+1}^{\alpha_{k_1+1}-1} \tag{4}$$

[6] where R_j describes the relative frequencies of variant type j in the population, $\bar{m}_j$ the observed absolute frequency in the sample $\bar{S}(t_1)$, $\bar{k}_1$ the number of variant types present in $\bar{S}(t_1)$ and α_j the Dirichlet exponents. In the following we assume $\alpha_j = \alpha = 1, \forall j$. Further, $R_{\bar{k}_1+1}$ denotes the aggregated relative frequency for all variant types not observed in the sample. We note that $[R_1, \ldots, R_{\bar{k}_1}, R_{\bar{k}_1+1}] * N_1$ with $[R_1, \ldots, R_{\bar{k}_1}, R_{\bar{k}_1+1}]$ sampled from Eq. (4) is an approximation of the population of cultural variants at t_1 which does not contain any temporal information (N_1 describes the estimated population size at time t_1). Therefore we derive the sampling pool at time t_1 by randomly drawing $w\nu(t_1)$ variants from this population under the constraint that all variant types present in the sample have to be present in the sampling pool as well. In each time step $t_1 + \tau$, $\tau = 1, \ldots, w$ we then remove $\nu(t_1 + \tau)$ cultural variants randomly and after at most w time steps all cultural variants in the sampling pool are characterized by their age and the removal procedure described above can be applied.

Given that the number of households and the sample sizes vary between phases, the number of production events and the number of cultural variants per production event will be phase- and time-dependent. In detail, we assume that the number of households evolve linearly over time

$$\bar{H}(t_i + \tau) = \bar{H}(t_i) + \frac{\bar{H}(t_{i+1}) - \bar{H}(t_i)}{\eta_i}\tau.$$

Further, the population size at time t_{i+1} is given by

$$\sum_{\tau=1}^{\eta_i} \rho_i \bar{H}(t_i + \tau) = N_{i+1}.$$

Then it holds

$$\sum_{\tau=1}^{\eta_i} \rho_i \bar{H}(t_i+\tau) = p_i \sum_{\eta=1}^{\eta_i} \left(\bar{H}(t_i) + \frac{\bar{H} t_{i+1}) - \bar{H}(t_i)}{\eta_i} \tau \right)$$
$$= \rho_i(\eta_i \bar{H}(t_i) + \frac{\bar{H}(t_{i+1}) - \bar{H}(t_i)}{\eta_i} \sum_{\tau=1}^{\eta_i} \tau = \rho_i \left(\eta_i \bar{H}(t_i) + (\bar{H}(t_{i+1}) - \bar{H}(t_i)) \frac{\eta_i + 1}{2} \right)$$
$$= \frac{\rho_i}{2} \left(\eta_i (\bar{H}(t_{i+1}) + \bar{H}(t_i)) + \bar{H}(t_{i+1}) - \bar{H}(t_i) \right).$$

It follows

$$\eta_i = \frac{\frac{2N_{i+1}}{\rho_i} - \bar{H}(t_{i+1}) + \bar{H}(t_i)}{\bar{H}(t_{i+1}) + \bar{H}(t_i)}$$

and consequently the number of production events is given by

$$\eta_i = \left[\frac{\frac{2N_{i+1}}{\rho_i} - \bar{H}(t_{i+1}) + \bar{H}(t_i)}{\bar{H}(t_{i+1}) + \bar{H}(t_i)} \right] \tag{5}$$

with $N_i = \bar{n}_i / r_i$ and [.] stands for rounding to the nearest integer. In this way we can specify population parameters for each phase separately. However, we note that we keep the parameter ρ, describing the number of cultural variants produced in each production event, and r, describing the fraction of the population of cultural variants that is recovered in the archaeological record, constant over time.

We carry out the simulation steps i. and ii. described above and generate T consecutive populations of size N_i. Lastly, we sample $\bar{n}_i$ cultural variants from each population in phase i conditioned on the assumed transmission process (characterized by the values of μ, b and w).

S1.3 Variable Population-Transmission Mode Version

Finally we allow both, population processes and cultural transmission processes, to vary over time. For this we consider each phases separately. Following the Dirichlet ansatz described in the last section, we generate a sampling pool at the beginning of each phase i and let η_i production events occur according to steps i. and ii. described above (η_i is calculated according to Eq. (5)). This produces a population of cultural variants at the end of phase i conditioned on the cultural transmission process characterized by the parameters b_i, μ_i and w_i. Drawing $\bar{n}_i$ variants from this population generates the theoretical sample of phase i.

S2 Appendix B - Statistical inference

In Appendix A we developed a modelling framework capturing the main population and cultural dynamics of the observed temporal changes in the pottery assemblage of the Merzbach region.

In particular, the cultural transmission process is described by the model parameters b_i, μ_i and w_i and in the following we use Bayesian inference techniques to determine the ranges of parameter values which are able to replicate the observed data up to an error level ε and therefore evaluate how accurately different transmission processes (and other model assumptions) can describe the observed archaeological assemblages Additionally we infer the values of the parameters r_i and ρ_i from the observed data as no external estimation procedures (i.e. estimation procedures based on other lines of evidence) can be found.

To do so we use approximate Bayesian computation (ABC) in the form of the rejection algorithm (see e.g. [7]) The aim of the ABC procedure is to approximate the (joint) posterior distributions of the model parameters, given the observed data, in situations where the likelihood function of the considered model is either impossible or computationally prohibitive to obtain (e.g. [7]). Broadly speaking, the posterior distributions of the parameters b_i, μ_i,w_i, r_i and ρ_i describe the parameter ranges which could have produced the observed data under a given error level ε.

S2.1 ABC rejection algorithm

In general, assuming that $\theta = [\theta_1, \ldots, \theta_p]$ model parameters of the simulation framework $\mathcal{M}$ are unknown and need to be inferred from the data the rejection algorithm is defined by the following steps.

i. Definition of prior distributions $p(\theta_1), \ldots, p(\theta_p)$ of the model parameters $\theta_1, \ldots, \theta_p$.

ii. Sampling of a vector $\tilde{\theta} = [\tilde{\theta}_1, \ldots, \tilde{\theta}_p]$ from the prior distributions.

iii. Producing simulation output by carrying out simulation $\mathcal{M}$ with the parameter values $\tilde{\theta}$ and if needed calculate summary statistics based on the output.

iv. Calculation of the Euclidean distance ε between the observed and simulated data.

v. Repetition of steps ii.-iv. for s times.

In our context we wish to infer the model parameters $\theta = [r_i, \rho_i, w_i, \mu_i, b_i]$ (see Tab. S1) as there exists no archaeological information that allows us to determine their values externally. Now on the base of the total s simulations of the framework described above we choose the 0.002% of the simulations that lead to the lowest error level and determine the posterior distributions for all model parameters. We note that it is crucial to examine the error level ε used produce the posterior distributions as its magnitude characterizes the accuracy of the approximation. If ε is large then this is an indication for a poor fit between theoretical and observed data and the assumed model might not be able to replicate the underlying cultural dynamic. This naturally raises the question of how the error level ε is calculated or in other words how theoretical and observed data are compared.

S2.2 Calculation of the error level ε

The observed data consists of the frequencies of cultural variant types at different points in time. Ideally we would like to compare theoretical and observed frequencies directly, however, this causes some difficulties for the equilibrium and variable-population version. The equilibrium version starts with a burn-in period and therefore there is no direct correspondence between the observed and simulated variant types.

In this case we could calculate the distance between theoretical and observed data in each phase i using the Simpson's diversity index

$$d_{\mathrm{div},i} = \sqrt{\left(\sum_{j=1}^{\bar{k}_i} \frac{\bar{s}_j(t_{i+1})}{\bar{n}_i} - \sum_{j=1}^{k_i} \frac{s_j(t_{i+1})}{\bar{n}_i}\right)^2}$$

and obtain $\varepsilon = \frac{1}{T-1} \sum_{i=1}^{T-1} d_{\mathrm{div},i}$. Alternatively, we re-order the observed and simulated variant types at the beginning of phase 1 according to their relative frequency and match the highest frequency with highest frequency, second-highest with second-highest and so on. We then track the frequencies of those variant types present at the beginning of phase 1 throughout all phases, calculate

$$d_{\mathrm{freq},i} = \sum_{j=1}^{\bar{k}_1} |\bar{s}_j(t_{i+1}) - s_j(t_{i+1})|$$

and subsequently $\varepsilon = \frac{1}{T-1} \sum_{i=1}^{T-1} d_{\mathrm{freq},i}$. We focus in the following on d_{freq} as analyses have shown that this statistic is more informative than d_{div}, i.e. produces a narrower posterior distribution ([7, 5]).

For the variable population and variable population-transmission mode versions we used the raw frequency based version of ε, only here the matching between observed and simulated variant types is naturally given. Focusing on the $\bar{k}_1$ variant types present at the beginning of the first phase allows for a straightforward calculation of the error tolerance and we see this choice justified as in the analyzed Merzbach dataset the sample observed in the last phase is made up to 80% of those $\bar{k}_1$ variant types. Additionally, in the variable popualtion-transmission mode version we can calculate $d_{\mathrm{freq},i}$ based on the variant types present at the beginning of each phase i.

S2.3 Prior distributions

The ABC framework described above requires prior distributions for all inferred model parameters. These distributions define the range of the parameter space which the parameters could possibly assume. Here most prior distributions (see Tab. S2 for the list of all parameters) were archaeologically informed (see section Prior and Constraints in the main text).

In particular, observations for the parameter w are given in calendar years, and converted into number of transmission events. In the equilibrium and variable population version this conversion is given by

$$w = \frac{\sum_{i=1}^{T} \eta_i}{Td} w_{\mathrm{y}}. \tag{6}$$

The parameter w_y defines the number of years that provide social information and d represents the estimated duration of each phase, which in this case was set to 20 years following [8]. In contrast, in the variable population-transmission mode version w is computed for each phase separately. Tab. S2 shows the values for each parameter which define the minimum and maximum of the parameter range of the uniform distribution. In addition $[17, 10, 12, 14, 15, 16, 12, 8]$ houses have been observed in the different phases.

**Symbol**	**Values**
b	$\mathcal{U}(0.5, 0.5)$
μ	$\mathcal{U}(0.001, 0.01)$
w_{y}	$\mathcal{U}(0.2, 5)$
ρ	$\mathcal{U}(1, 5)$
r	$\mathcal{U}(0.25, 0.75)$

Table S2: Prior distributions of the inferred parameters

S3 Appendix C - Inference Results

In this Appendix we provide the results of the full analysis of the equilibrium, variable population and variable population-transmssion-mode versions.

S3.1 Posterior Distributions

Marginal and joint posterior distributions (Fig. S2–S10) have been computed sampling the parameter values of the 1,000 simulations with lowest ε out of 50 million simulations (corresponding to 0.002 %). In all instances the 95% highest posterior density interval is shown in black and the parameter w_y (instead of w) is shown in order to ensure comparability.

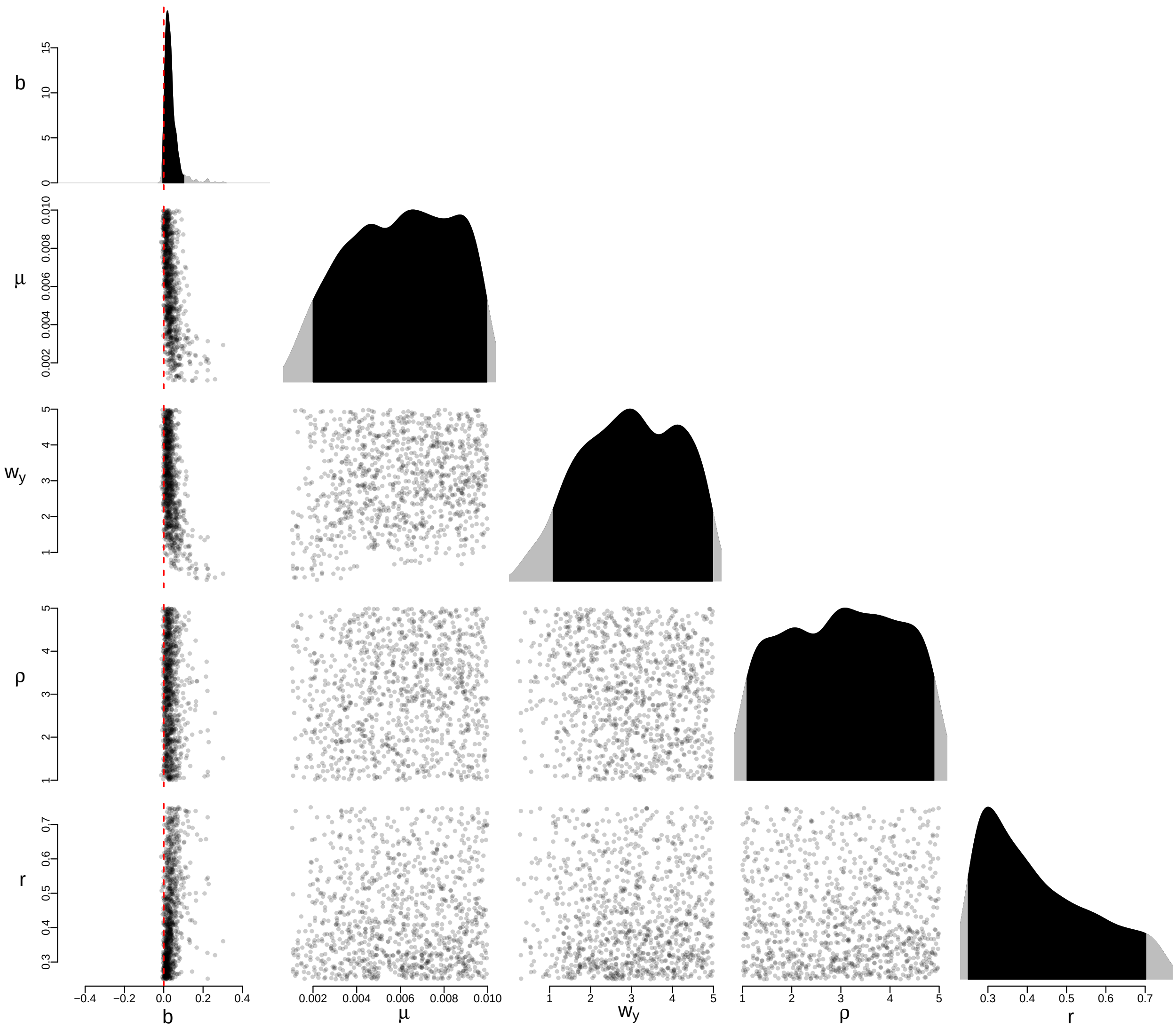

Figure S2: Marginal and joint posterior distributions of the equilibrium version.

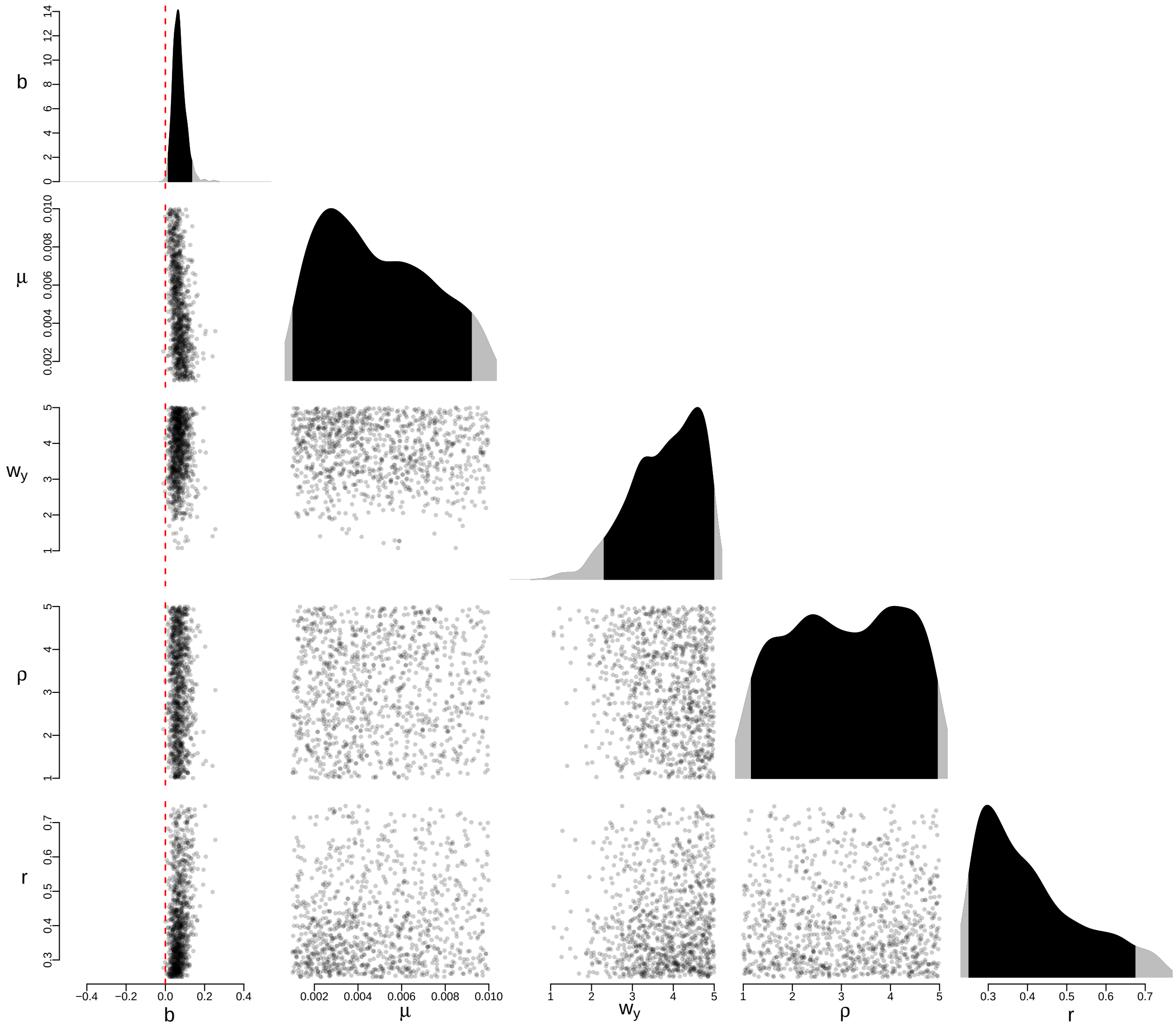

Figure S3: Marginal and joint posterior distributions of the variable population version.

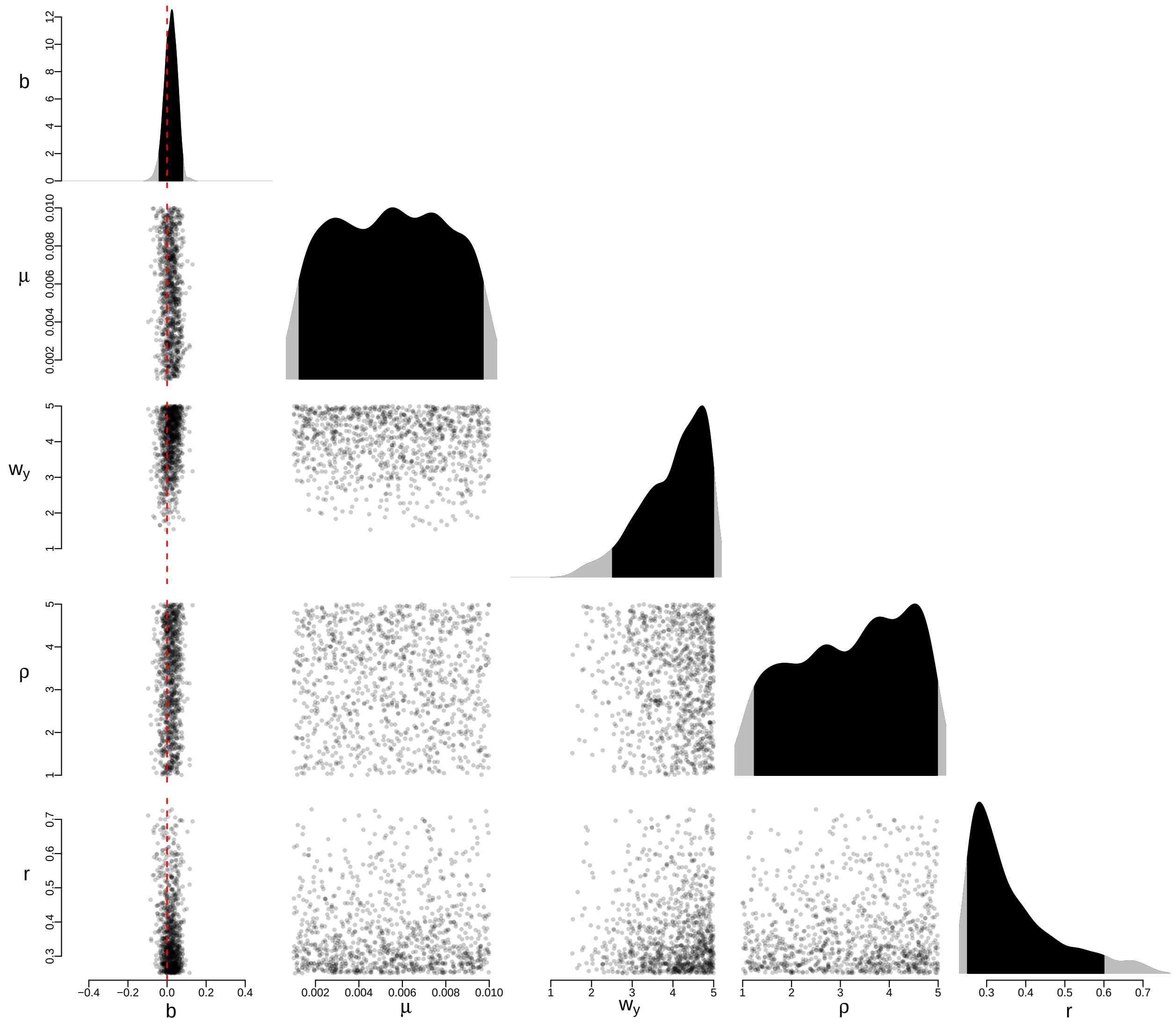

Figure S4: Marginal and joint posterior distributions of the variable population-transmission mode version for phase VIII.

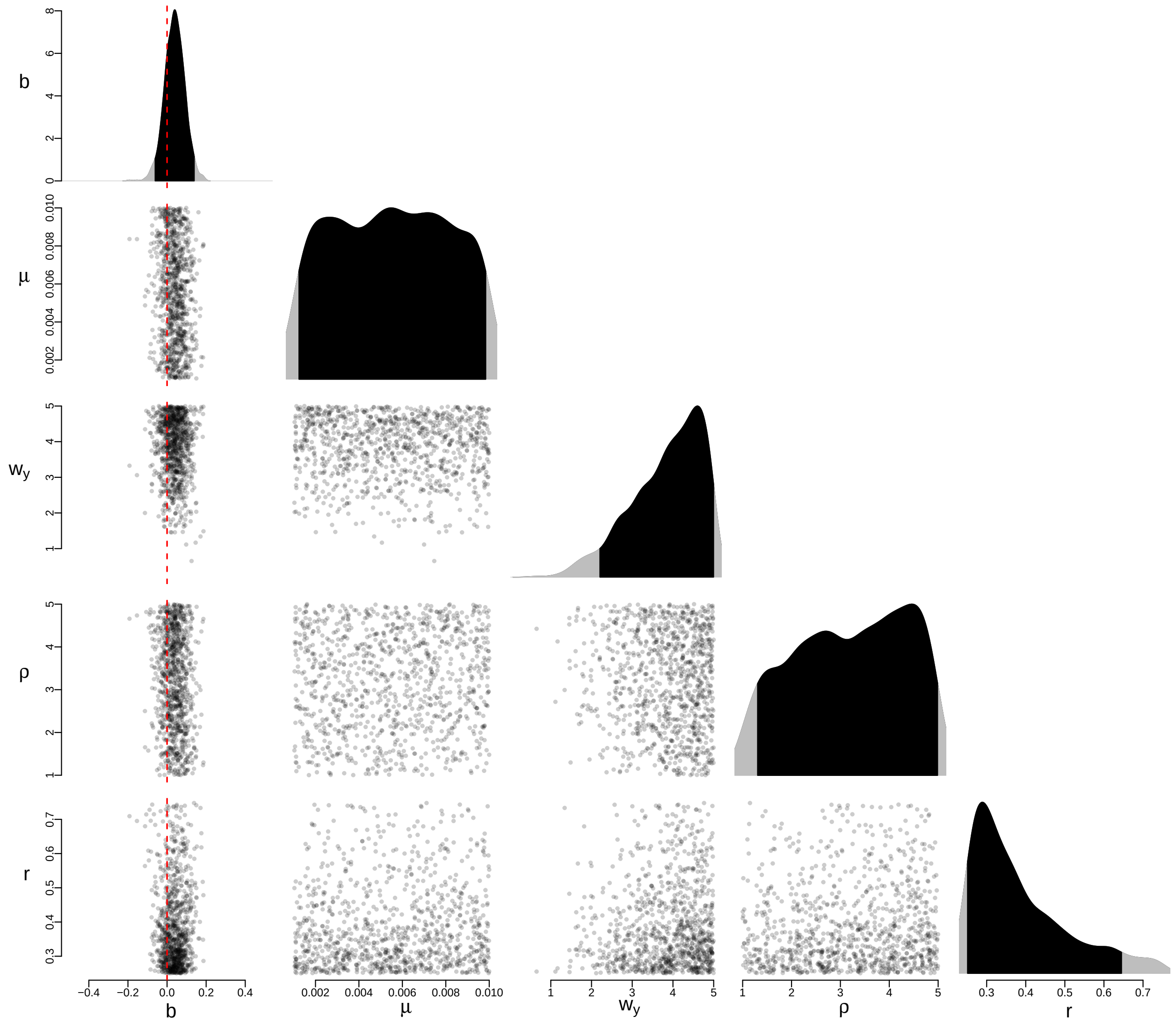

Figure S5: Marginal and joint posterior distributions of the variable population-transmission mode version for phase IX.

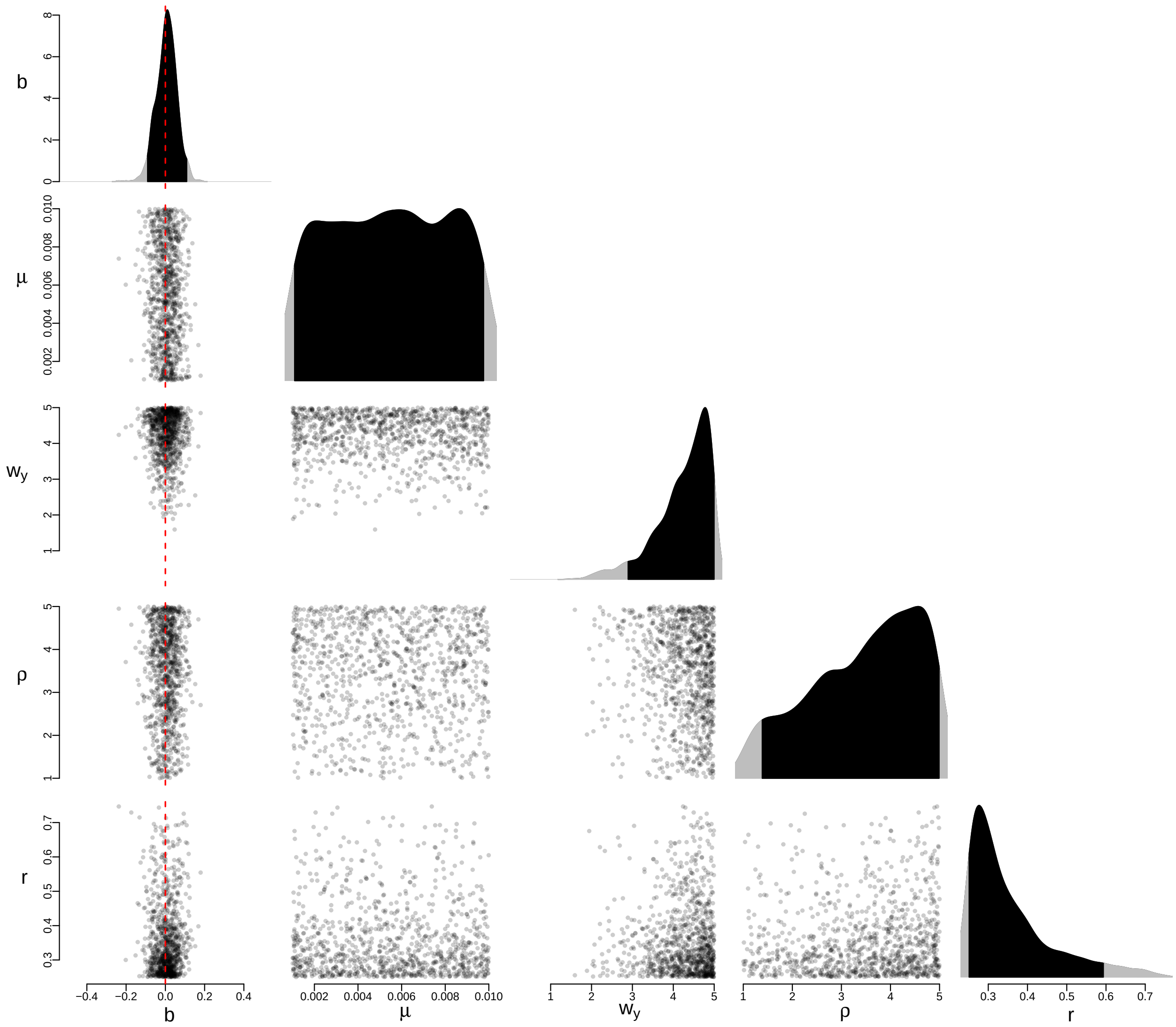

Figure S6: Marginal and joint posterior distributions of the variable population-transmission mode version for phase X.

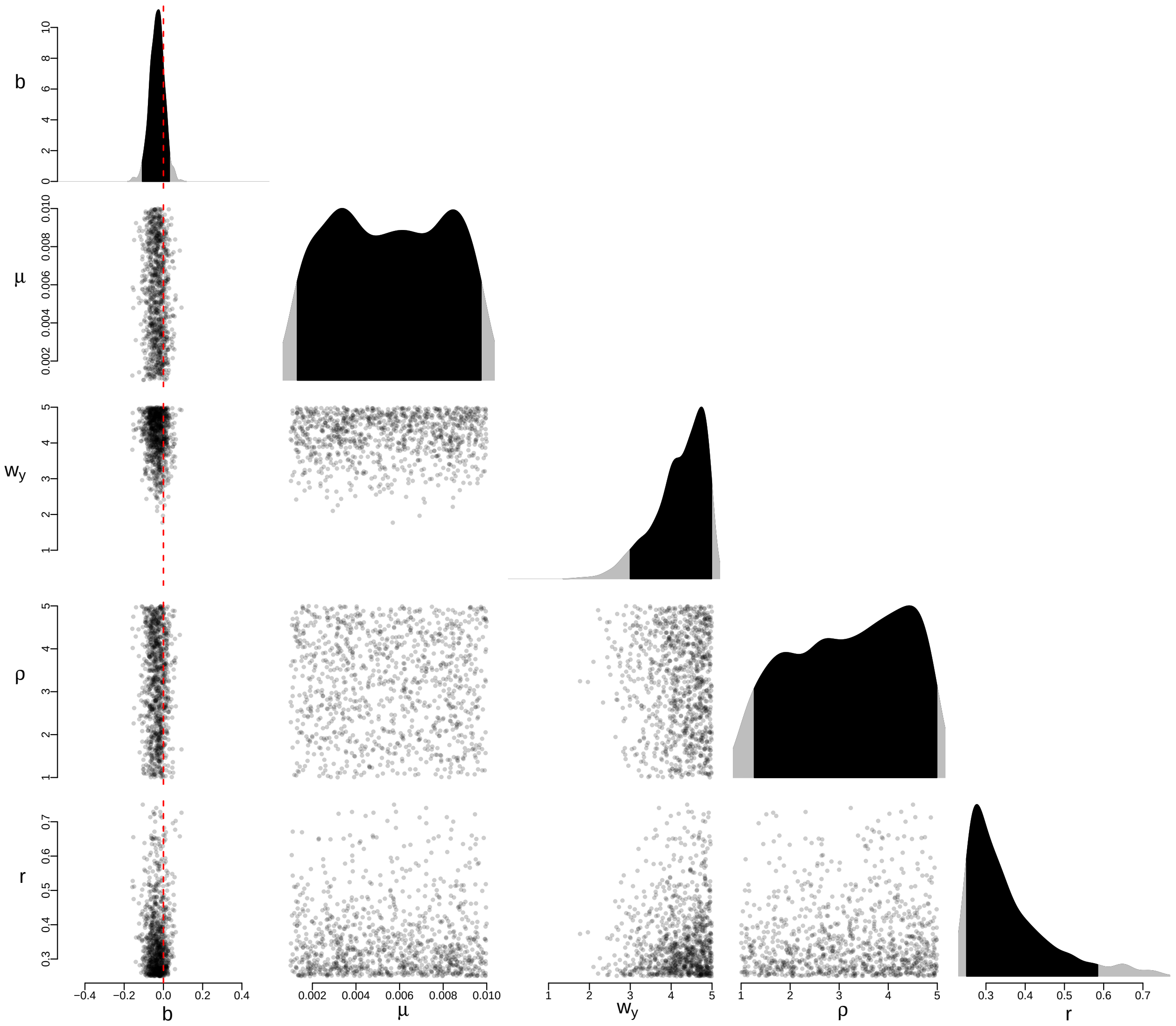

Figure S7: Marginal and joint posterior distributions of the variable population-transmission mode version for phase XI.

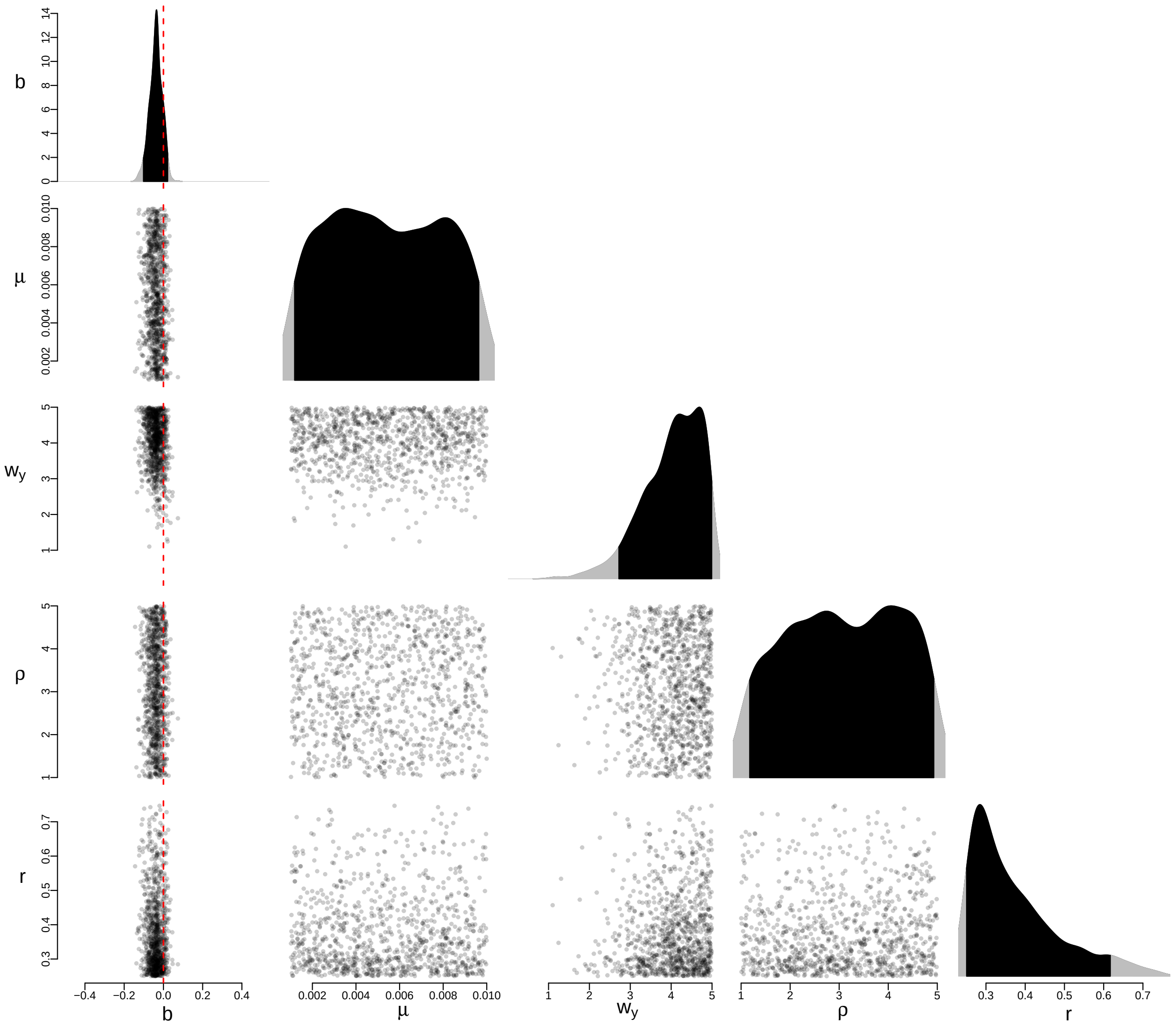

Figure S8: Marginal and joint posterior distributions of the variable population-transmission mode version for phase XII.

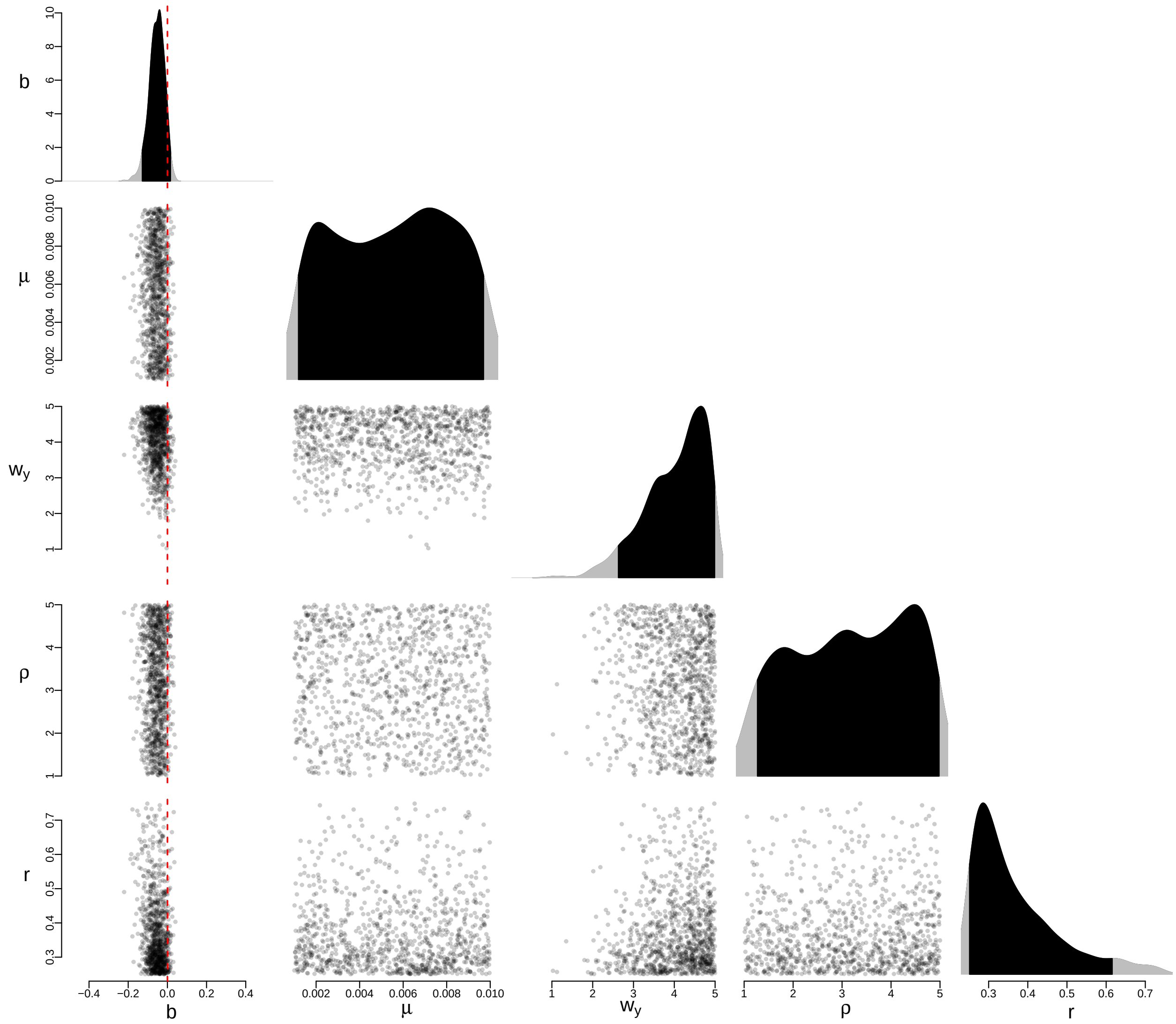

Figure S9: Marginal and joint posterior distributions of the variable population-transmission mode version for phase XIII.

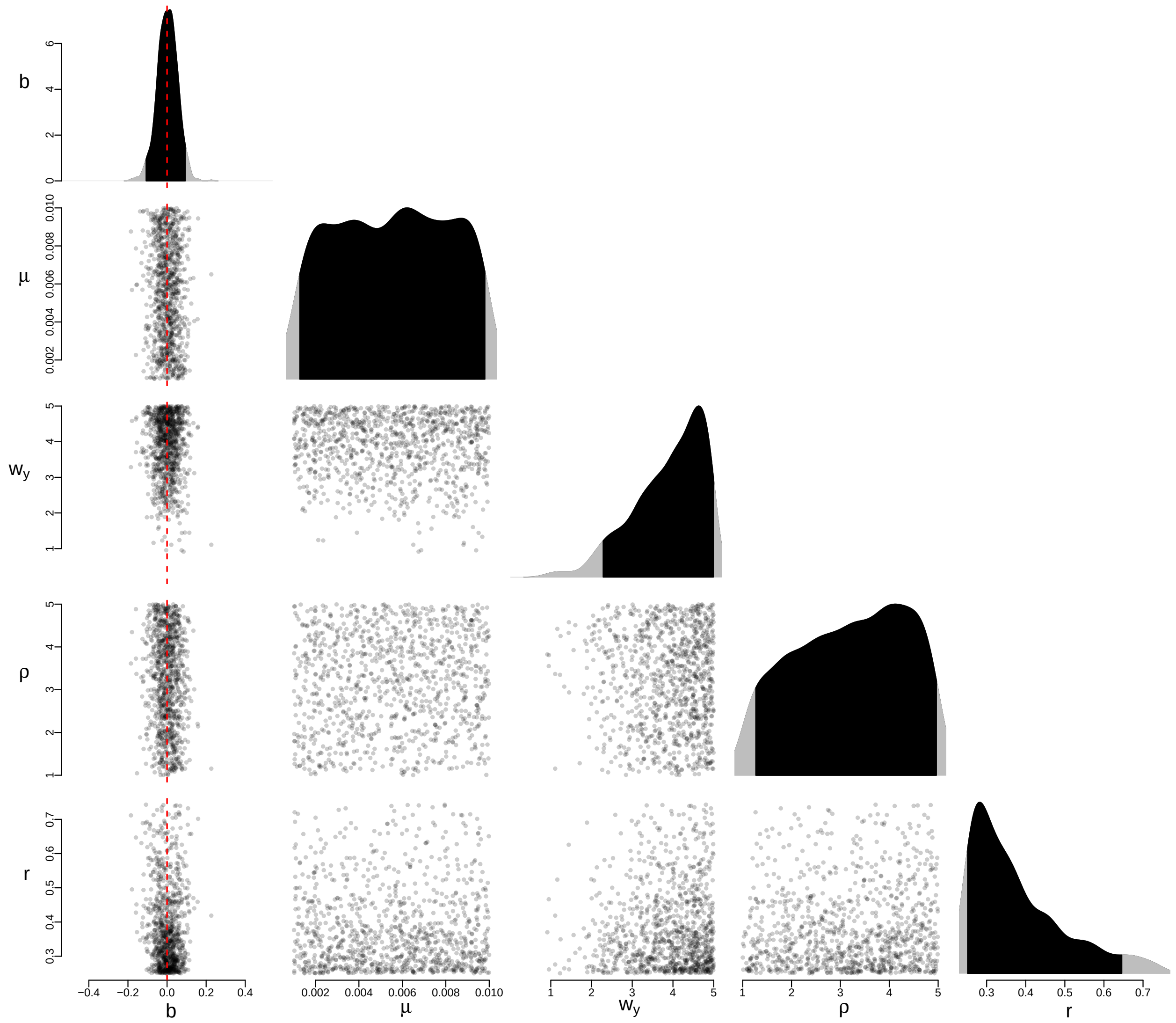

Figure S10: Marginal and joint posterior distributions of the variable population-transmission mode version for phase XIV.

S3.2 Posterior Predictive Checks

Posterior predictive checks (Figs. S11–S13) have been computed by re-running the simulations with the parameter settings of the joint posterior distribution and recording the frequencies of the cultural variants for each phase. The shaded area in Figs. S11 and S12 and the solid lines in Fig. S13 represent the 95% HPDI of the variant frequencies.

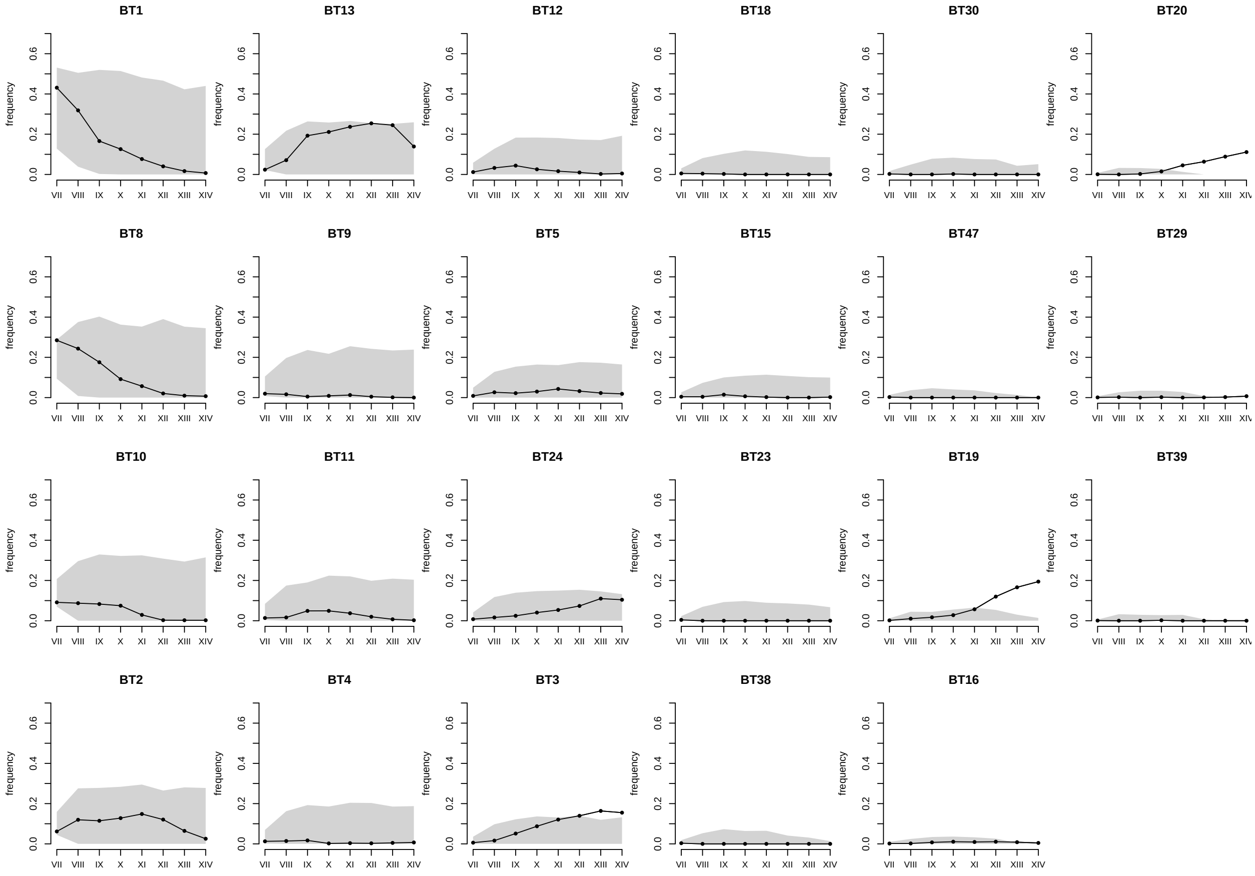

Figure S11: Posterior predictive check for the equilibrium version. The 95% HPDI is shown as grey areas, whilst the observed frequencies as dotted lines.

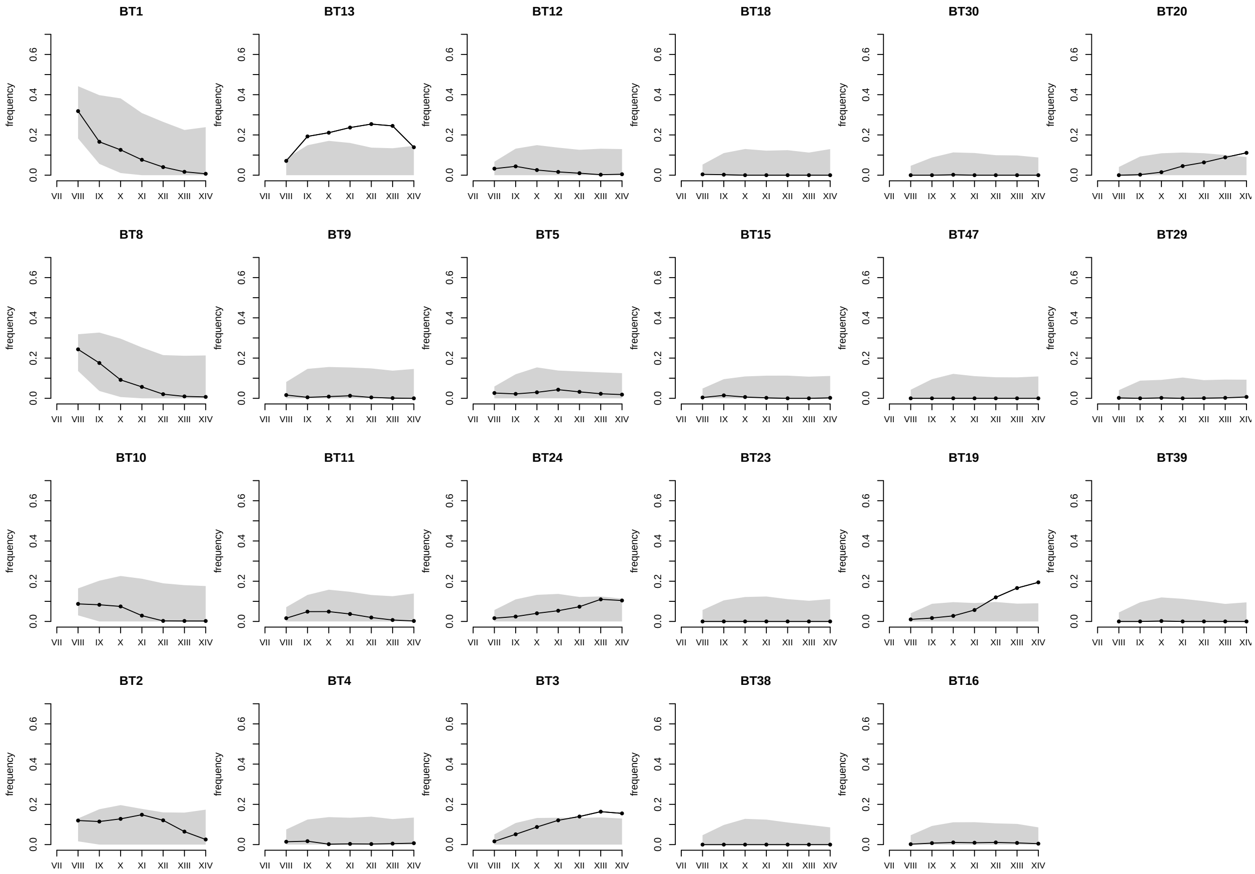

Figure S12: Posterior predictive check for the variable population version. The 95% HPDI is shown as grey areas, whilst the observed frequencies as dotted lines.

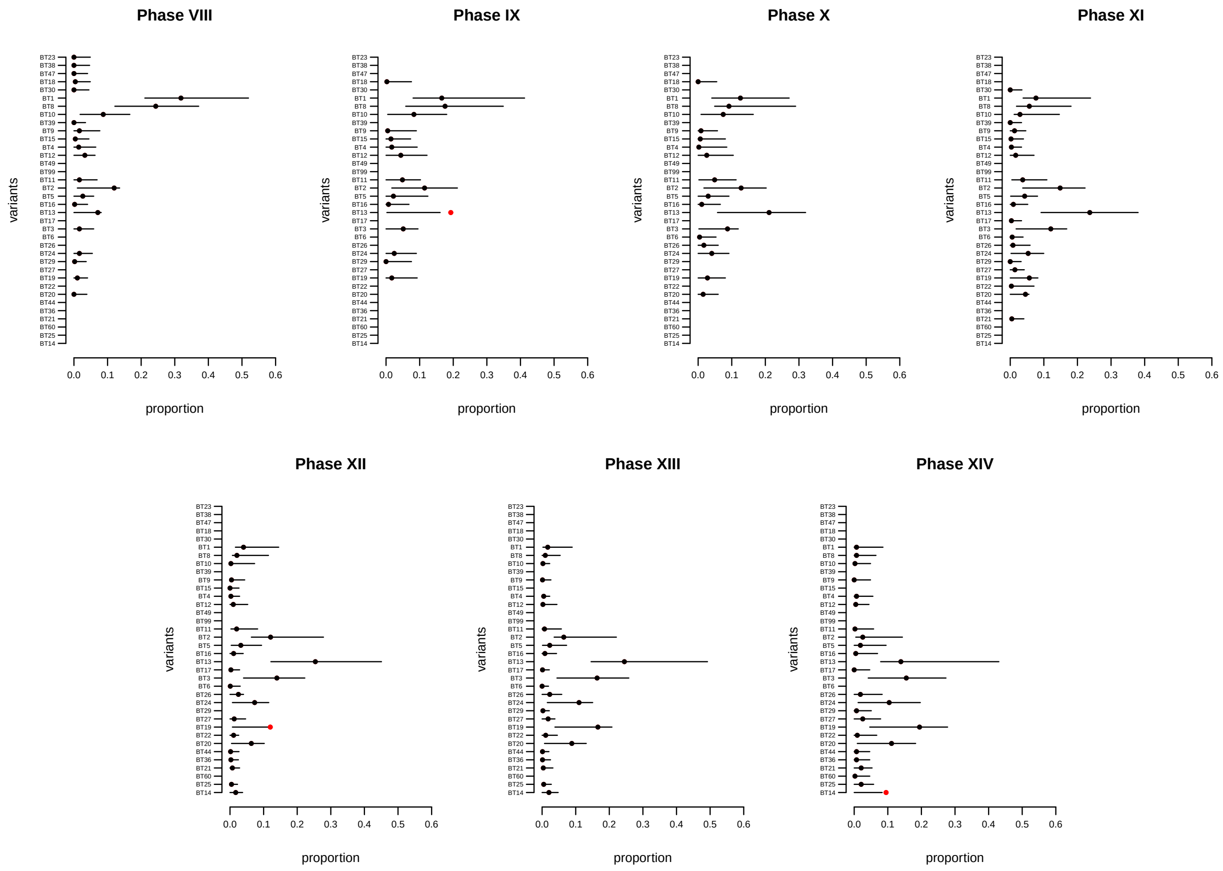

Figure S13: Posterior predictive check for the variable population- transmission mode version. Observed frequency within 95% HPDI are shown as black dots, whilst those outside the range are shown as red dots.

S3.3 Overlap Analysis

In order to quantify the changes in the process of cultural transmission between phases we calculate the area of overlap between the posterior distributions of the selection strength b (see [9, 10] for details of the calculation). The area of overlap is 1 if two distributions are identical and 0 if the distributions cover non-overpapping parameter regions. Therefore, the smaller the area of overlap the more different are the processes of cultural transmission in the respective phases.

		**Equilibrium**	**Var.Pop.**	**Var.Pop./Trans.**						
				**VIII**	**IX**	**X**	**XI**	**XII**	**XIII**	**XIV**
**Equilibrium**		-	-	-	-	-	-	-	-	-
**Var.Pop.**		0.282	-	-	-	-	-	-	-	-
**Var.Pop./Trans.**	**VIII**	0.793	0.407	-	-	-	-	-	-	-
	**IX**	0.647	0.628	0.748	-	-	-	-	-	-
	**X**	0.619	0.374	0.814	0.737	-	-	-	-	-
	**XI**	0.224	0.086	0.413	0.376	0.596	-	-	-	-
	**XII**	0.168	0.047	0.361	0.327	0.539	0.883	-	-	-
	**XIII**	0.105	0.032	0.289	0.268	0.461	0.78	0.808	-	-
	**XIV**	0.566	0.35	0.763	0.71	0.94	0.646	0.592	0.513	-

Table S3: Area of overlap between two posterior distributions of the selection strength b for different phases.

S3.4 Error Levels

Distribution of the error levels (ε) of the best 0.002% of the simulations has been retrieved for each version of the model and normalised to allow a comparison between the different versions of the model (i.e. ε of the equilibrium and the variable population versions have been divided by the 8 and 7, the respective number of phases compared between observed and simulated data in each case; see Fig. S14).

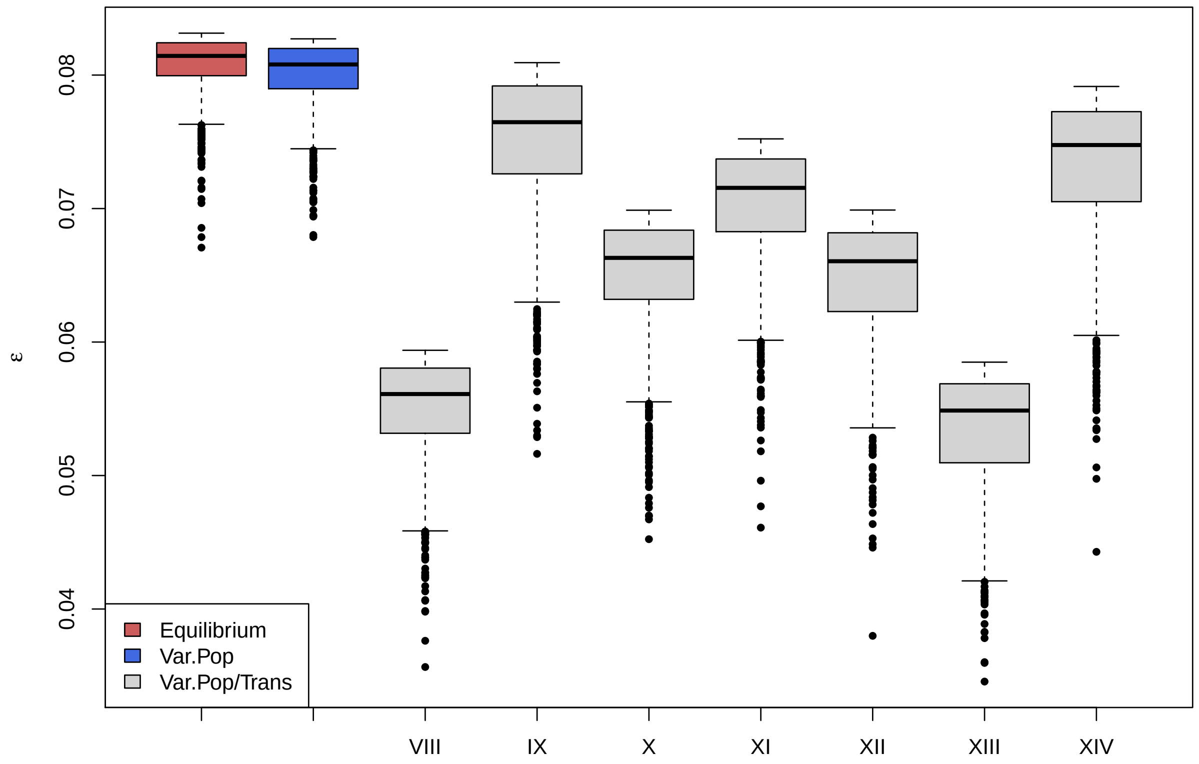

Figure S14: Per-phase error levels (ε) of the three versions

References

[1] K. N. Laland, "Social learning strategies," *Learning and Behavior*, vol. 32, pp. 4–14, 2004.

[2] F. D. Neiman, "Stylistic Variation in Evolutionary Perspective: Inferences from Decorative Diversity and Interassemblage Distance in Illinois Woodland Ceramic Assemblages," *American Anitquity*, vol. 60, no. 1, pp. 7–36, 1995.

[3] R. Boyd and P. J. Richerson, *Culture and the Evolutionary Process*. Chicago: University of Chicago Press, 1985.

[4] E. R. Crema, K. Edinborough, T. Kerig, and S. J. Shennan, "An Approximate Bayesian Computation approach for inferring patterns of cultural evolutionary change," *Journal of Archaeological Science*, vol. 50, pp. 160–170, Oct. 2014.

[5] A. Kandler and S. Shennan, "A generative inference framework for analysing patterns of cultural change in sparse population data with evidence for fashion trends in LBK culture," *Journal of The Royal Society Interface*, vol. 12, p. 20150905, Dec. 2015.

[6] A. Gelman, J. B. Carlin, H. S. Stern, and D. B. Rubin, *Bayesian data analysis*, vol. 2. Chapman & Hall/CRC Boca Raton, FL, USA, 2014.

[7] M. A. Beaumont, W. Zhang, and D. J. Balding, “Approximate Bayesian Computation in Population Genetics,” *Genetics*, vol. 162, pp. 2025–2035, Dec. 2002.

[8] J. Lehmann, “Die Keramik und Befunde des bandkeramischen Siedlungsokatzes Erkelenz-Kckhoven, Kreis Heinsberg (Grabungskampagnen 1989-1994),” in *Der bandkeramische Siedlungsplatz von Erkelenz-Kckhoven I: Archlogie* (H. Koschik, ed.), pp. 1–364, Mainz: P. von Zabern, 2004.

[9] K. Kura, M. Broom, and A. Kandler, “Modelling dominance hierarchies under winner and loser effects,” *Bulletin of mathematical biology*, vol. 77, no. 6, pp. 927–952, 2015.

[10] B. Wilder and A. Kandler, “Inference of cultural transmission modes based on incomplete information,” *Human biology*, vol. 87, no. 3, pp. 193–204, 2015.
